# *Trichinella spiralis* galectin disrupts gut epithelial integrity and mediates larval invasion via acting MUC13- ROCK2/MAPK pathway

**DOI:** 10.1371/journal.pntd.0014684

**Published:** 2026-09-21

**Authors:** Ru Zhang, Yao Zhang, Xin Zhuo Zhang, Pei Kun Cong, Jin Yi Wu, Shao Rong Long, Ruo Dan Liu, Jia Xu, Jing Cui, Zhong Quan Wang

**Affiliations:** 1 Department of Parasitology, School of Basic Medical Sciences, Zhengzhou University, Zhengzhou, China; 2 School of Basic Medical Science, Putian University, Key Laboratory of Translational Tumor Medicine in Fujian Province, Putian, China; George Washington University, UNITED STATES OF AMERICA

## Abstract

**Background:**

Gut epithelium serves as a critical barrier against *Trichinella spiralis* larval invasion, parasite-derived galectins are involved in host–parasite interactions, including invasion and immune modulation. This study investigated the role of *T. spiralis* galectin (Tsgal) in gut epithelial barrier disruption and larval invasion.

**Methodology/principal findings:**

Recombinant Tsgal (rTsgal) was expressed *in vitro* and its interaction with intestinal epithelial cells (IEC) was ascertained using immunofluorescence test (IFT) and confocal microscopy. The rTsgal-specific binding IEC proteins were identified by GST pull-down, mass spectrometry (MS) and Co-immunoprecipitation (Co-IP) assay. Intestinal epithelial integrity damage induced by rTsgal was evaluated by IFT, qPCR and Western blotting analysis. The *in vitro* larva invasive assay was also conducted. The results showed that rTsgal bound and interacted specifically with MUC13 receptor in Caco-2 cells. rTsgal activated MUC13-ROCK2/MAPK pathway, reduced the expression of gut epithelial tight junctions (TJs; ZO-1, E-cad, Occludin and Claudin-1), increased gut epithelial permeability, disrupted gut epithelial integrity and barrier functions, thereby facilitated larva invasion of Caco-2 monolayers. Moreover, MUC13 receptor inhibitor (Cuc D), MUC13 knockdown of Caco-2 cells, ROCK2 inhibitor (KD025) and p38-MAPK inhibitor (SB203580) suppressed this pathway activation, restored gut epithelial integrity, and impeded larval invasion.

**Conclusions:**

Tsgal disrupted gut epithelial integrity and mediated larval invasion through binding to MUC13 receptor and activating ROCK2/MAPK pathway. Tsgal might be a potential target molecule for development of novel drugs and vaccines to block *T. spiralis* infection.

## Author summary

Intestinal epithelium is a crucial barrier to defense *Trichinella spiralis* larva invasion, but the mechanism of larval penetration of gut epithelium remains unclear. Galectin is widely distributed in parasites, and plays a significant act in the invasion process and immunomodulation. Our study aimed to reveal the mechanism of *T. spiralis* galectin (Tsgal) mediating larval invasion of intestinal epithelium. The results of GST pull-down, mass spectrometry (MS) and Co-IP showed that rTsgal bound and interacted specifically with MUC13 receptor in Caco-2 cells. Trans-epithelial electrical resistance (TEER), gut epithelial paracellular permeability, qPCR and Western blot analysis showed that rTsgal activated MUC13-ROCK2/MAPK pathways, reduced expression levels of gut epithelial tight junctions (TJs; ZO-1, E-cad, Occludin and Claudin-1), increased paracellular permeability and damaged intestinal epithelial integrity, and mediated larva invasion. Moreover, MUC13 receptor inhibitor (Cuc D), MUC13 knockdown of Caco-2 cells, ROCK2 inhibitor (KD025) and p38-MAPK inhibitor (SB203580) inhibited the activation of ROCK2/MAPK pathway, they also abrogated and reduced the rTsgal role on disrupting enteric TJs integrity and barrier function, and impeded larva invasion. Our findings indicated that Tsgal played a critical role in destroying enteric barrier integrity and mediating larva invasion. These insights refined our understanding for *T. spiralis* larval invasion mechanism and offered a novel potential target for anti-*T. spiralis* vaccine.

## Introduction

*Trichinella spiralis* is a nematode parasitized various animals; its adults and larvae colonize respectively in the gut epithelium and skeletal muscles of infected mammals. Humans usually get *Trichinella* infection through eating infected meat that has not been cooked properly [1]. *T. spiralis* pathogenicity is higher than other *Trichinella* species due to its higher female fecundity (newborn larval production of each female adult worm) and the stronger immune reaction in humans. The nematode’s settlement in host small intestine marks the beginning of the pivotal intestinal phase, which is represented by increased number of goblet cells, mucin secretion and an inflammatory infiltration [2]. Intestinal epithelial cells (IEC) function as the natural barrier against larval invasion and the first responders that contact with parasites [3]. *T. spiralis* intestinal infectious larval (IIL) invasion of intestinal epithelium is a critical step to establish the nematode infection, and understanding larval invasion mechanism is valuable to develop new drugs and anti-*Trichinella* vaccine [4,5].

The gut barrier is the first line defense against invading enteric pathogens. This barrier comprises intestinal epithelial cells connected by the tight junctions (TJs), and is protected by a mucous layer secreted by goblet cells [6]. Some pathogens secrete enzyme-like substances, which degrade mucus or disrupt the TJs among gut epithelial cells, thereby damaging gut integrity [7]. *Giardia* possesses the ability to destroy mucin, an *in vitro* experiment indicated that of *Giardia lamblia* trophozoite adhesion to Caco-2 cells damaged the TJs [8,9]. Early studies indicated that serine proteases and cysteine proteases in *T. spiralis* excretory/secretory proteins (ESPs) compromised gut epithelial integrity by degrading TJs, playing a pivotal role in the parasite’s invasion, growth, and survival in host [10]. When *T. spiralis* C-type lectin binds to syndecan-1 in Caco-2 cells, the STAT3 pathway is activated, gut TJs expression is reduced, gut epithelial integrity is compromised, and *T. spiralis* invasion is mediated [11]. As a result, there are probably many parasite target molecules for vaccine exploitation from the standpoint of parasite-host interactions.

Galectins belong to a family of S-type lectins; they are characterized by a carbohydrate recognition domain (CRD) and specific affinity for β-galactoside [12]. Recent studies revealed that galectins act a key function in inflammation, immune response, cellular migration processes and signal cascades. Galectin-3 binds to the MUC1 extracellular domain through the galectin-3 C-terminal CRD and its N-terminal ligand multi-merization domain, promoting EGFR dimerization and activation in human epithelial cancer cells [13]. The CRD of some parasite-derived galectins exhibits pathogen-associated molecular patterns (PAMP)-like activity and recognizes Toll-like receptors in host cell [14,15]. Binding and interaction of *E. histolytica* elongation factor 1 alpha (EF1a) and intermediate subunit of lectin (Igl) modulate the phagocytosis [16]. The findings showed that galectins derived from parasites play an active role in interactions between parasites and mammalian hosts. They may also play a part in parasite invasion and infection [17].

A beta-galactoside-binding lectin (Tsgal; GenBank: XM_003381608.1) from *T. spiralis* was discovered in our earlier research [18]. The Tsgal is the tandem repeat (TR) type galectin and had two CRD sequence motifs binding to β-galactoside [19]. Tsgal was expressed at diverse *T. spiralis* worm stages, mainly localized in the cuticle and stichosome of this parasite. Tsgal specially combined with IEC and promoted larval intrusion into IEC, and anti-rTsgal antibodies inhibited larva intrusion, Vaccination of mice with rTsgal elicited a systemic mixed Th1/Th2 response, and a significant immune protection against *T. spiralis* infection [20,21]. rTsgal drove macrophages to polarize towards M1 and enhances the ability of macrophages killing the NBL [22]. Furthermore, rTsgal induced intestinal inflammation, activated the ERK/NF-κB pathway, selectively interacted with intestinal epithelium TLR-4 receptor, and participated in larva penetration of gut mucosa [23]. The results suggested that Tsgal interacted with IEC and destroyed gut epithelium barrier, and mediated larva invasion at the process of early *T. spiralis* infection. However, the mechanism of larval disrupting the gut epithelial integrity is unclear at present. Thus, the purpose of this research was to ascertain if Tsgal compromises the integrity of gut epithelium and facilitates the invasion of *T. spiralis* larvae, to further characterizes the key protein molecule and its acting mechanisms in the larval invasion process.

## Materials and methods

### Ethics and statement

This research was carried out in accordance with the National Guidelines for Experimental Animal Welfare (Minister of Science and Technology, People’s Republic of China, 2006). All animal experimental protocol in this research was approved by the Life Science Ethics Committee of Zhengzhou University (ZZUIRB GZR 82272367).

### *Trichinella*, cell and animal

The species of *Trichinella* used in this experiment was the *T. spiralis* isolate (ISS534) which was collected from a naturally infected pig in Nanyang City, Henan Province, China [24]. Human colon adenocarcinoma cell line (Caco-2) used in this study were derived from our laboratory’s cell bank. Female BALB/c mice with 4–6 weeks old were procured from the Experimental Animal Center of Zhengzhou University.

### Preparation of *T. spiralis* soluble somatic antigens

At 42 days post infection (dpi), *T. spiralis* muscle larvae (ML) were obtained from infected mouse skeletal muscles by artificial digestion [25]. To obtain the IIL, each mouse was further administered orally with 5000 ML, and killed at 6 hours after infection. The ML and IIL worms’ soluble protein (SP) were made in accordance with earlier publications [26].

### Preparation of recombinant Tsgal (rTsgal) and anti-rTsgal serum

Full-length sequence of Tsgal gene (GeneBank: XM_003381608.1) was cloned, and recombinant pQE-80L/Tsgal was transferred into the *Escherichia coli* BL21 (DE3) (Novagen, USA). In this research, 0.5 mM IPTG was used to induce rTsgal expression for 6 hours at 37 °C [18]. By using Ni-NTA-Sefinose resin (Sangon Biotech Co., Shanghai, China), the rTsgal with a His-tag was purified. GST-Sepharose Resin 4FF (Settled Resin) (Beyotime, Shanghai, China) was also used to purify GST-tagged rTsgal in our laboratory [23].

A combination of 20 μg rTsgal emulsified with complete Freund’s adjuvant was administered subcutaneously to each of twenty BALB/c mice. After that, three booster vaccinations with 20 μg rTsgal emulsified with incomplete Freund’s adjuvant were administered every two weeks. Blood samples were obtained following the final vaccination, immune sera were isolated and anti-rTsgal IgG titer was assayed by ELISA with rTsgal as coated antigen, and the IgG titer was 1:10^5^ [27].

### Cell culture and CCK-8 assay

The Caco-2 cells were cultivated at 37°C with 5% CO_2_ in DMEM (Servicebio, Wuhan, China) replenished with 1% non-essential amino acids (Solarbio, Beijing, China), 10% fetal bovine serum (FBS, VivaCell, Shanghai, China), 100 U/ml penicillin and 100 µg/ml streptomycin [28]. In order to assess the rTsgal’s effect on cellular viability, six different concentrations of rTsgal (10, 20, 30, 40, 50 and 100 µg/ml) diluted in serum-free culture medium were added to the culture plates and incubated for 6 and 12 h. The plate was then incubated for one hour after 10 μl of CCK-8 solutions (Epizyme Biotech, Shanghai, China) were added to each well [29,30]. A plate reader (Tecan, Switzerland) was served to assay the absorbance at 450 nm. Cell viability was calculated relative to the DMEM control group. Cell viability was calculated as a percentage by normalizing the absorbance of treated cells versus DMEM-only controls. Blank values were subtracted from both measurements to account for background signal.

### Interaction of rTsgal and Caco-2 cell assayed by Immunofluorescence test (IFT) and confocal microscopy

The IFT was conducted to assess the interaction of rTsgal and Caco-2 cells as previously described [31]. Briefly, Caco-2 cell on coverslips were incubated at 37 °C for 2 h with 50 μg/ml rTsgal, IIL SP antigens and PBS, respectively. The cells were fixed using 4% paraformaldehyde for 20 min, permeabilized with 0.1% (v/v) Triton X-100 for 10 min, blocked with 1% bovine serum albumin (BSA) at 37 °C for 1 h, and then incubated with 1:10 dilutions of corresponding primary antibodies (anti-rTsgal serum, infection serum and pre-immune serum) at 37 °C for 1 h. After being washed with PBS, coverslips were added with appropriate fluorescence-conjugated secondary antibodies (Alexa Fluor 488-conjugated anti-mouse IgG, 1:100 dilution; Abways, Shanghai, China). Then, cell coverslips were re-stained with 4′,6-diamidino-2-phenylindole (DAPI) for 5 min. The fluorescence signal were examined under confocal microscope (Olympus, Tokyo, Japan) [32].

### GST pull-down test

To further certify the kind of specific Caco-2 cell proteins that binds to rTsgal, we conducted a GST pull-down test [11]. The GST-rTsgal fusion protein was immobilized on glutathione-Sepharose 4B beads and subsequently incubated with whole-cell lysates of Caco-2 cells at 4 °C for 4 h to allow complex formation. Following capture of the protein conjugates, the bound complexes were denatured and analyzed by SDS-PAGE and Western blotting [33]. Control experiments included GST alone to assess background binding specificity.

### LC/MS-MS and bioinformatic analysis

As previously mentioned, an approximately 260 kDa Caco-2 cell protein band binding with rTsgal was detected by the GST-pull down test, was removed from the gel and used to in-gel tryptic digestion [34]. Following digestion, tandem MS analysis and high-performance liquid chromatography (HPLC) were employed to separate the peptide mixtures. All LC/MS/MS spectrum data were searched by MaxQuant 2.4.14.0. All protein sequences were annotated to the Gene Ontology (GO) identifiers using interproscan (version: 5.52-86.0) with default parameters. GO functional categorization and statistical analysis were performed using the ‘topGO’ software [35]. The molecular weight (MW) and isoelectric point (pI) of related protein were then subjected to quantitative statistics.

### Molecular docking of Tsgal and MUC13

The amino acid sequences of Tsgal (GenBank: XP_003381656.1) and MUC13 (GenBank: NP_149038.3) was obtained from the NCBI website (https://www.ncbi.nlm.nih.gov/gene). Three-dimensional (3D) structures of MUC13 were obtained by SWISS MODEL (https://swissmodel.expasy.org/). Then, the docking of Tsgal and MUC13 was executed with HDOCK (http://hdock.phys.hust.edu.cn/). HDOCK is a web-based platform dedicated to protein–protein, protein–DNA, and protein–RNA molecular docking. It uses ITScorePP to assess the binding modes and a fast Fourier transform (FFT)-based algorithm to globally sample all potential binding poses. A global blind docking mechanism is used, and all docking parameters are set to default values [36,37]. The 3D structure of the complex model and the amino acid residues within a distance of 5 Å interaction in the two proteins were optimized using PYMOL [38].

### Confocal microscopy of co-localization of rTsgal and MUC13

Caco-2 cells were seeded on glass coverslips and Caco-2 monolayer was treated with 50 μg/ml rTsgal at 37 °C for 2 h. Then, the monolayer was fixed with 4% paraformaldehyde for 20 min. The monolayers were blocked with 1% BSA at 37 °C for 1 h after permeabilization and three PBS washes. Subsequently, the monolayer was probed by primary antibodies: anti-rTsgal serum (1:20) and anti-MUC13 antibody (1:100; Proteintech, Wuhan, China) overnight at 4°C. Alexa Fluor 488-conjugated goat anti-mouse IgG (1:100; Abways, Shanghai, China) and CY3- conjugated goat anti-rabbit IgG (1:200; servicebio, Wuhan, China) were served as the secondary antibody. Cell nuclei were counterstained blue by DAPI for 10 min. The slides were washed three times with PBS and imaged by a confocal microscopy [39,40]. The Pearson’s R correlation coefficient was used to quantify the fluorescent co-localization index of Caco-2 cells between the respective channels, which was performed using ImageJ software [41].

### Co-immunoprecipitation (Co-IP) assay

Co-IP proteins were extracted from cultured cells incubated with rTsgal, Protein A/G Plus-agarose beads (Santa cruz, America) were added and conjugated with antibodies against mouse-IgG and Caco-2 lysates at 4 °C for 1 h to pre-clear protein lysates. The above-lysates and anti-rTsgal antibody were incubated with fresh beads at 4 °C on a rotating device for 4 h. Then, the beads were washed with DISC lysis buffer (30 mM pH8.0 Tris-HCl, 120 mM NaCl, 10% glycerol, 1% TritonX-100), soaked in lysis buffer and eluted by boiling in loading buffer. Immunoprecipitated proteins were subjected to immunoblot analysis for the protein of interest. Anti-rTsgal and anti-MUC13 antibodies were used as antibody for Co-IP [42,43].

### Total RNA extraction and real-time quantitative PCR (qPCR)

To assess the MUC13’ role in rTsgal-activated ROCK2/MAPK pathways, a Caco-2 monolayer was treated with 1 μM Cucurbitacin D (Cuc D, a MUC13 inhibitor) for 24 h, and then stimulated with rTsgal for 2 h [44]. For qPCR analysis of mRNA expression levels of MUC13, ROCK2 and TJs (ZO-1, E-cad, Occludin and Claudin-1), Trizol reagent was used to extract the total RNAs from treated Caco-2 cells as previously reported [17,32], then converted to cDNA using Multiplex One Step RT-PCR Kit (Yeasen, Shanghai, China), according to the previous protocol. An SYBR green mix (Servicebio, Wuhan, China) was used for PCR reaction on an Applied Biosystems 7500 Fast System (Life Technologies, USA) and primer sequences indicated in S1 Table. Glyceraldehyde-3-phosphate dehydrogenase (GAPDH) was served as a housekeeping gene and the relative mRNA expression was analyzed using the 2^−ΔΔCt^ method [22,38].

### Western blot analysis

The intestinal epithelial barrier is the host’s earliest line of resistance invading pathogens. TJs proteins are an important component of intestinal barrier. We investigate whether rTsgal regulates gut epithelial TJs expression. Western blot analysis was executed as before [45,46]. Concisely, Caco-2 cells were homogenized in ice-cold extraction buffer (Beyotime, Shanghai, China) and cell soluble proteins were harvested. An equal quantity of protein was separated using SDS-PAGE. It was then transferred onto a polyvinylidene difluoride (PVDF; Millipore, Sigma, USA). Following being blocked by 5% nonfat dried milk in Tris-buffered saline with 0.05% Tween (TBST), the primary antibody against ZO-1 (1:1000, Servicebio, China), E-cad (1:40000, Abcam, UK), Occludin (1:500, Invitrogen, USA), Claudin-1 (1:250, Invitrogen, USA), ROCK2 (1:1000, Abways, Shanghai, China), p38-MAPK (1:1000, Abmart, Shanghai, China), p-p38-MAPK (1:1000, Abmart, Shanghai, China), Tubulin (1:5000, Abmart, Shanghai, China) and GAPDH (1:1000, Servicebio, China) were added and incubated overnight at 4 °C on a shaker. Then, the membranes were incubated at 37 °C for 1 h with HRP-conjugated secondary antibodies (goat anti-rabbit IgG diluted at 1:10000; Sangon, Shanghai, China). After washes with TBST, the reacted bands were colored with Omni-ECL reagents (Epizyme, Shanghai, China), images were captured using a chemiluminescence image system (Tanon, China), and analyzed with ImageJ Software [34,47].

### The *in vitro* assay of trans-epithelial electrical resistance (TEER) and FITC-Dextran permeability

Caco-2 cells (4 × 10^4^ cells/well) were seeded on the 0.4 μm pore transwell inserts (BIOFIL, China) and maintained for 21 d. The TEER values of Caco-2 monolayer were assayed using a Millicell-ERS volt-ohmmeter (Millipore, USA). The resistance value, measured in ohms (Ω), was acquired by subtracting the TEER value of the blank insert and multiplying the difference by the growth surface area of the filter. Then, paracellular permeability of Caco-2 monolayer was ascertained with 4 kDa FITC-dextran (FD-4, 0.5 mg/ml, Sigma, USA) [24]. PBS was added to the lower chamber. FD-4 was dissolved in DMEM and added to the top chamber [48,49]. After incubation for 2 h, 200 μl of the solution was collected from the lower chamber and the fluorescent intensity was determined using a microplate reader at 485–520 nm (Tecan, Switzerland).

### Transient RNAi knockdown MUC13 gene in Caco-2 cells

For transient knockdown MUC13 gene, transfection of Caco-2 cells with MUC13 siRNAs (Sangon, Shanghai, China) was performed using RNATransMate reagent (Sangon, Shanghai, China) according to the manufacturer’s instructions. Three pairs of sequences were listed as follows: siNC sense: 5′-UUCUCCGAACGUGUCACGU-3′, antisense: 5′-ACGUGACACG UUCGGAGAA-3′, siMUC13#1 sense: 5′- CUCAGCUGAUGCUGUAACA-3′, antisense: 5′-UGUUACAGCAUCAGCUGAG-3′, siMUC13#2 sense: 5′-CGACUGUAAGGACAAA UUUCA-3′, antisense: 5′-UGAAAUUUGUCCUUACAGUCG-3′ [50–52]. Transfection procedures began once Caco-2 monolayers achieved 70% confluence. Following incubation at 37 °C for 6 h, transfection efficiency was evaluated via fluorescence microscopy. Successful transfection was confirmed when 80% of cellular populations displayed intense green fluorescent signals. Subsequently, culture medium was replaced with complete media, and cells were maintained for an additional 48-hour period. Knockdown efficiency at both mRNA and protein levels was confirmed by qPCR and Western blot analysis.

The original images of all gels and Western blots were included in S1 Raw Images.

### The *in vitro* larval invasive test

To evaluate whether MUC13 receptor and ROCK2/MAPK pathway modulate rTsgal’s promotion effect on larva invasion of Caco-2 monolayer, an *in vitro* larva intrusion assay was conducted as mentioned earlier [22]. Concisely, Caco-2 monolayer was pretreated with rTsgal, Cuc D, siMUC13, KD025 or SB203580. After being exposed to 5% swine bile for two hours at 37 °C, the ML were first activated into the IIL. Next, 50 IIL were added onto Caco-2 monolayer in DMEM semisolid media (DMEM with L-glutamine, 15 mM HEPES, and 1.75% agarose). Larva intrusion of monolayer was examined and counted under a microscope following 2 hours of cultivation at 37 °C with 5% CO_2_. The invaded larvae are defined as motile larvae that actively penetrate and migrate within Caco-2 monolayer, leaving clear migratory traces within the gel structure. In contrast, non-invaded larvae are those remaining coiled on the surface of the monolayer without evidence of penetration or migration. The percentage of invaded parasites to the total number of parasites seen in each test was used to calculate the larva invasion (%) [53].

### Statistical analysis

The data were statistically analyzed with the aid of IBM SPSS Statistics 21.0 software. The data were presented as mean ± standard deviation (SD). Data distribution and variance homogeneity were assessed using the Shapiro–Wilk test and Levene’s test, respectively. For comparisons among multiple groups, one-way analysis of variance (one-way ANOVA) followed by Fisher’s least significant difference (LSD) post-hoc test was performed when a significant overall group effect was observed. For comparisons between two groups, an independent-sample Student’s t-test was used for normally distributed data with equal variances, whereas Welch’s t-test was applied when variances were unequal. The Chi-square test was applied to analyze the differences of percentage of invaded larvae among experimental groups. All experiments were repeated at least 3 times in this study. A value of *P* < 0.05 was considered statistically significant.

## Results

### Effect of rTsgal on Caco-2 cellular viability

To evaluate rTsgal impact on Caco-2 cellular survival, cells were incubated with rTsgal (0, 10, 20, 30, 40, 50 and 100 μg/ml) for 6 or 12 h, and cellular viability was assessed by CCK-8 assay. Six-hour incubation did not produce any cytotoxicity at 10–100 μg/ml concentrations (*F* = 2.853, *P* > 0.05), whereas 12-hour incubation with 30, 40, 50, and 100 μg/ml rTsgal significantly compromised cellular survival (S1 Fig), indicating that rTsgal had a time-dependent cytotoxic effect. Based on these assays, 50 μg/ml rTsgal and incubation for 2 h were adopted to avoid cytotoxic interference in subsequent experiments.

### Binding and cellular localization of rTsgal and Caco-2 cells

To characterize the interaction and localization of rTsgal with Caco-2 cells, IFT with laser scanning confocal microscopy were employed. Following exposure to rTsgal, intense green fluorescent signals were observable on Caco-2 cells when probed with anti-rTsgal serum and infection sera. Additionally, the predominant binding site of rTsgal and Caco-2 was specifically located in cell membrane (S2 Fig). The observations collectively demonstrated the capacity of rTsgal binding to Caco-2 cellular surfaces.

### GST pull-down identification of interaction protein between rTsgal and Caco-2 cells

**Fig 1:**
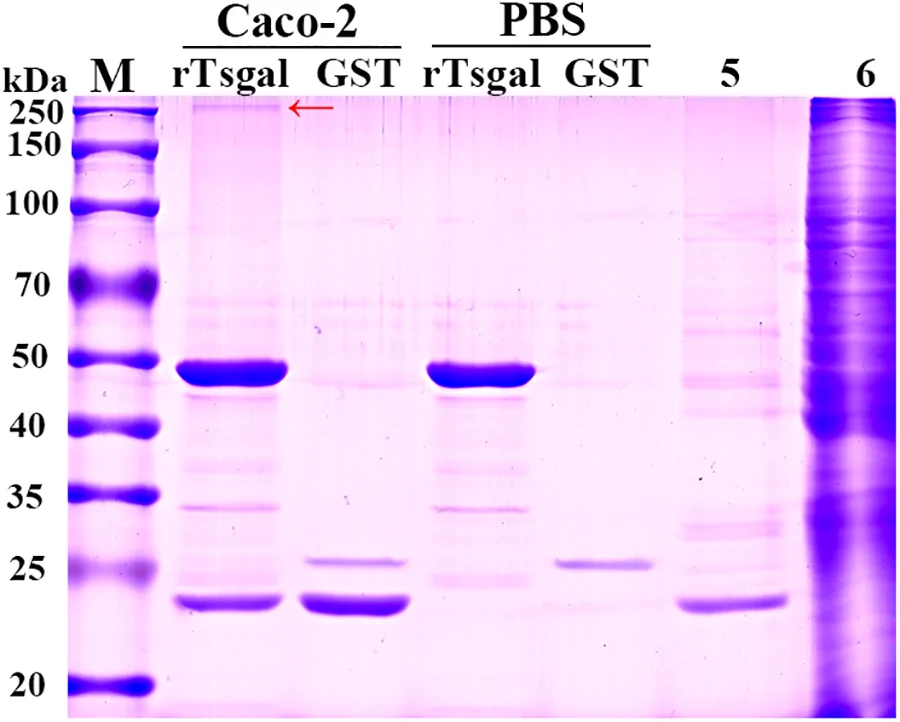
Binding of rTsgal and Caco-2 by GST pull down test. rTsgal was co-incubated with the Caco-2 protein lysates to capture the interacting proteins; M: Marker; Lane 5: GSH-conjugated resin after incubation with Caco-2 protein lysates; Lane 6: Caco-2 protein lysates.

The results of GST pull-down test revealed that a clear band of approximate 260 kDa was observed in the rTsgal lane co-incubated with Caco-2 lysates (as indicated by the red arrow, Fig 1), while no corresponding band was observed in the GST-tag lane, suggesting that the captured protein band might be the particular interaction proteins of rTsgal and Caco-2 cell**.**

### Mass spectrometry, GO annotation and theoretical two-dimensional distribution of rTsgal bound-Caco-2 cell proteins

Potential binding proteins of rTsgal-Caco-2 cells were identified and listed in Table 1. They are LY75, CDHR2, EL52 (HSP90α), TLN1, MUC13 and ENO2. To systematically elucidate the functional attributes of these candidates, Gene Ontology terms were hierarchically classified into biological process (BP), cellular component (CC) and molecular function (MF). The analysis of the MW and pI values of the interaction proteins exhibited that the MW of these proteins were widely distributed, mainly ranging from 50 to 200 kDa, and the pI values were mainly in the range of 5–9 (S3 Fig). The coverage rate of MUC13 is as high as 6%. MUC13 is a glycoprotein, highly and constitutively expressed in small and large intestines, indicating that MUC13 was probably interacted with rTsgal and helped control the production of TJs protein in intestinal epithelium. Hence, in this study, MUC13 was selected for the subsequent experiments.

**Table 1:** LC/MS-MS analyzed the interaction proteins between rTsgal and Caco-2 cells captured by GST pull-down.

Protein names	Protein ID	Gene name	PI	MW [kDa]	Pipetids	Coverage (%)	GO
Lymphocyte antigen 75	O60449	LY75	6.67	198.2	14	10	defense response; response to stress; immune response; transpor; endocytosis; establishment of localization; vesicle-mediated transport; inflammatory response; biological process.
Cadherin-related family member 2	Q9BYE9	CDHR2	4.5	141.5	1	1	cellular process; response to stimulus; biological regulation.
Heat shock protein HSP 90-alpha	K9JA46	EL52	6.67	84.6	5	1	immune system process; metabolic process; cellular process; signaling; multicellular organismal process; response to stimulus; localization.
Talin-1	Q9Y490	TLN1	6.07	269.6	1	0	cytoskeleton organization; cell-cell adhesion; response to stress; regulation of body fluid levels; cell junction assembly; epithelial structure maintenance; cell communication; cellular response to stimulus; cell surface receptor signaling pathway.
Mucin 13 (MUC13)	Q9H3R2	MUC13	5.07	54.6	2	6	multicellular organismal process; biological regulation.
phosphopyruvate hydratase	F5H0C8	ENO2	4.87	34.7	1	4	reproduction; metabolic process; cellular process; reproductive process; multicellular organismal process; regulation of biological process; stimulus; biological regulation.

The blue font in the Table 1 represents the screened rTsgal-interacted Caco-2 cell MUC13 protein which is further characterized in this study.

### rTsgal binding with MUC13

**Fig 2:**
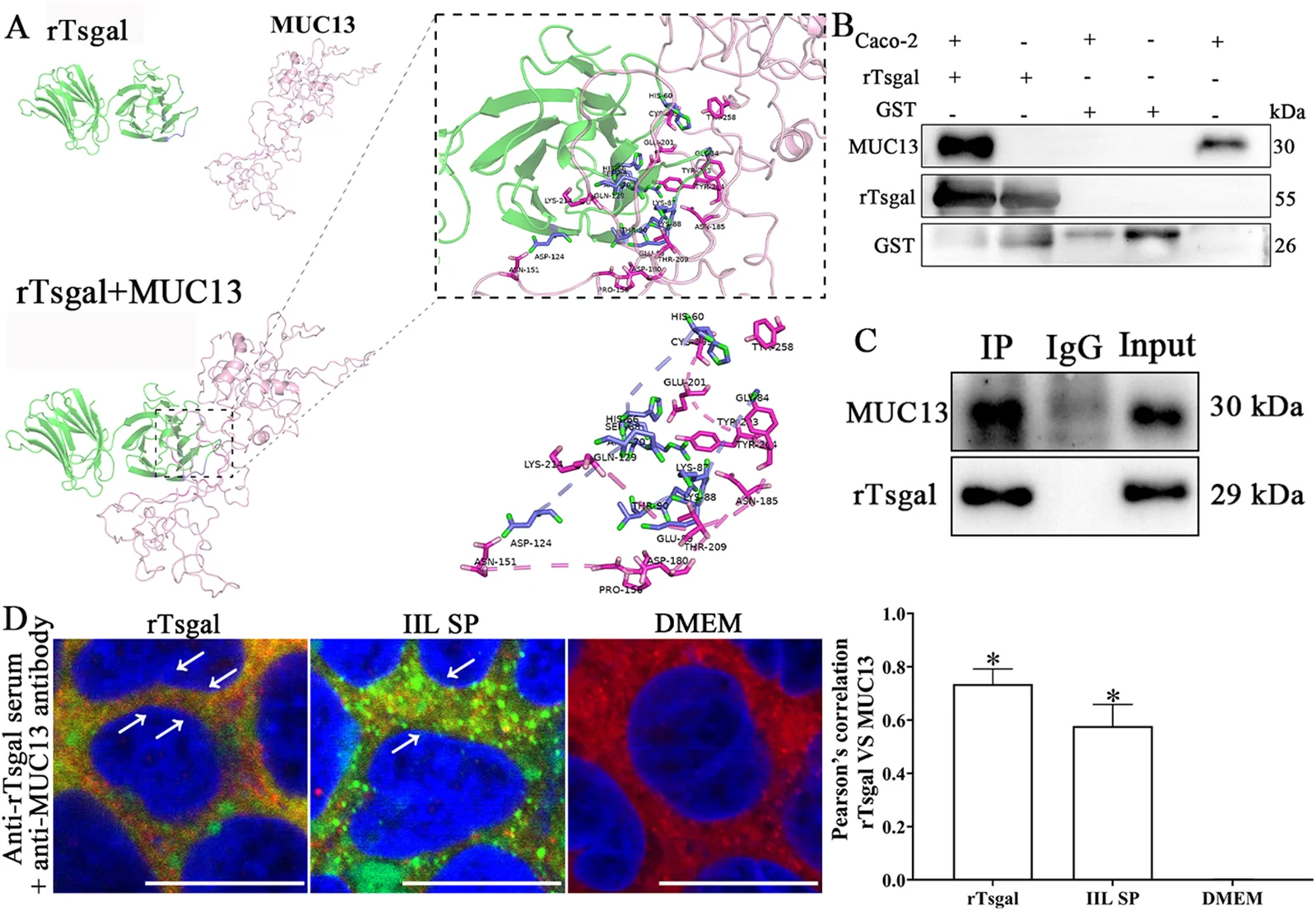
rTsgal binds wiht MUC13 in Caco-2 cells. **A:** Modeled complex structure between rTsgal (green, left) and MUC13 (purple, right). The hydrogen bond is shown by dashed lines. On the right, it was the enlarged image for the interface in the catalytic site. **B:** GST pull-down assay showing the interaction between rTsgal and MUC13**.** Caco-2 cell lysates was mixed with GST-rTsgal or GST tag, and then immobilized on glutathione Sepharose beads. After washing, the eluted proteins were subjected to Western blotting analysis with anti-rTsgal and anti-MUC13 antibodies, respectively. **C:** Co-IP analyses were performed to investigate the binding and interaction between rTsgal and MUC13 in Caco-2 cells. IP: Co-IP complex (rTsgal and MUC13 from Caco-2 cell proteins); IgG: normal mouse IgG; Input: Caco-2 cellular MUC13 protein after being co-cultured with rTsgal. **D:** IFT analyses of co-localization of rTsgal and MUC13 in Caco-2 cells by using anti-rTsgal (green) and anti-MUC13 (red) antibodies. Yellow showed double-stained rTsgal and MUC13 (As indicated by white arrows). Quantitative co-localization analysis between rTsgal and MUC13 was performed by using ImageJ-produced Pearson’s correlation coefficients. Three independent experiments were performed. Data were analyzed using one-way ANOVA, ^*^P < 0.05 compared to the DMEM group. Scale bars: 25 μm.

To testify the binding and interaction between rTsgal and MUC13, we performed molecular docking first. S2 Table presents the molecular docking scores of interaction between rTsgal and MUC13. The lowest binding score for the docking of rTsgal and MUC13 is -233.12, indicating a strong binding affinity between the two proteins. As shown in Fig 2A, there are hydrogen bond interactions between the amino acid residues near the interaction interface of rTsgal and MUC13. The molecular docking results suggested the binding of rTsgal to MUC13. To further testify the binding and interaction of rTsgal and MUC13, the mutational analysis of galectin CRD or MUC13 was performed in this study. Several residues within the CRD region of rTsgal were predicted to participate in this interaction. To investigate the role of CRD region for binding with MUC13, three predicted key residues (His60, Arg70 and Lys87) were individually mutated to alanine (Ala). Compared with the wild-type rTsgal–MUC13 complex, the docking score of the mutant rTsgal (M-rTsgal)–MUC13 complex was reduced from −330.83 to −260.12. Simultaneously, three predicted interaction residues (Tyr203, Tyr204 and Lys214) of MUC13 were individually mutated to alanine; the docking score of rTsgal- M-MUC13 complex was reduced from -330.83 to -241.52, compared with rTsgal–MUC13 complex (S2 Table). The results showed that when the M-rTsgal or M-MUC13 was used, their predicted binding affinity was clearly declined, suggested that the CRD region of rTsgal contributes to the interaction with MUC13. Furthermore, a GST pull-down assay using purified GST-rTsgal validated this interaction *in vitro* (Fig 2B). To further ascertain whether rTsgal binds with MUC13, Co-IP assay was conducted. The findings showed that agarose beads coupled with anti-rTsgal serum successfully precipitated the protein complex of rTsgal and MUC13. However, MUC13 and rTsgal were not captured by normal mouse IgG (Fig 2C). The results of IFT verified the binding and interaction between rTsgal and MUC13 (S4 Fig). When Caco-2 cells were incubated with rTsgal, more binding of rTsgal and MUC13 was colocalized in the cytomembrane, compared to the DMEM group (Fig 2D). The Pearson’s R correlation coefficient of rTsgal group was obviously higher than the DMEM group (*F* = 137.582, *P* < 0.001). Taken together, our data strongly suggested that rTsgal bound to and interacted with MUC13, with its binding region located in the cell membrane.

### rTsgal reduced intestinal epithelial TJs expression

**Fig 3:**
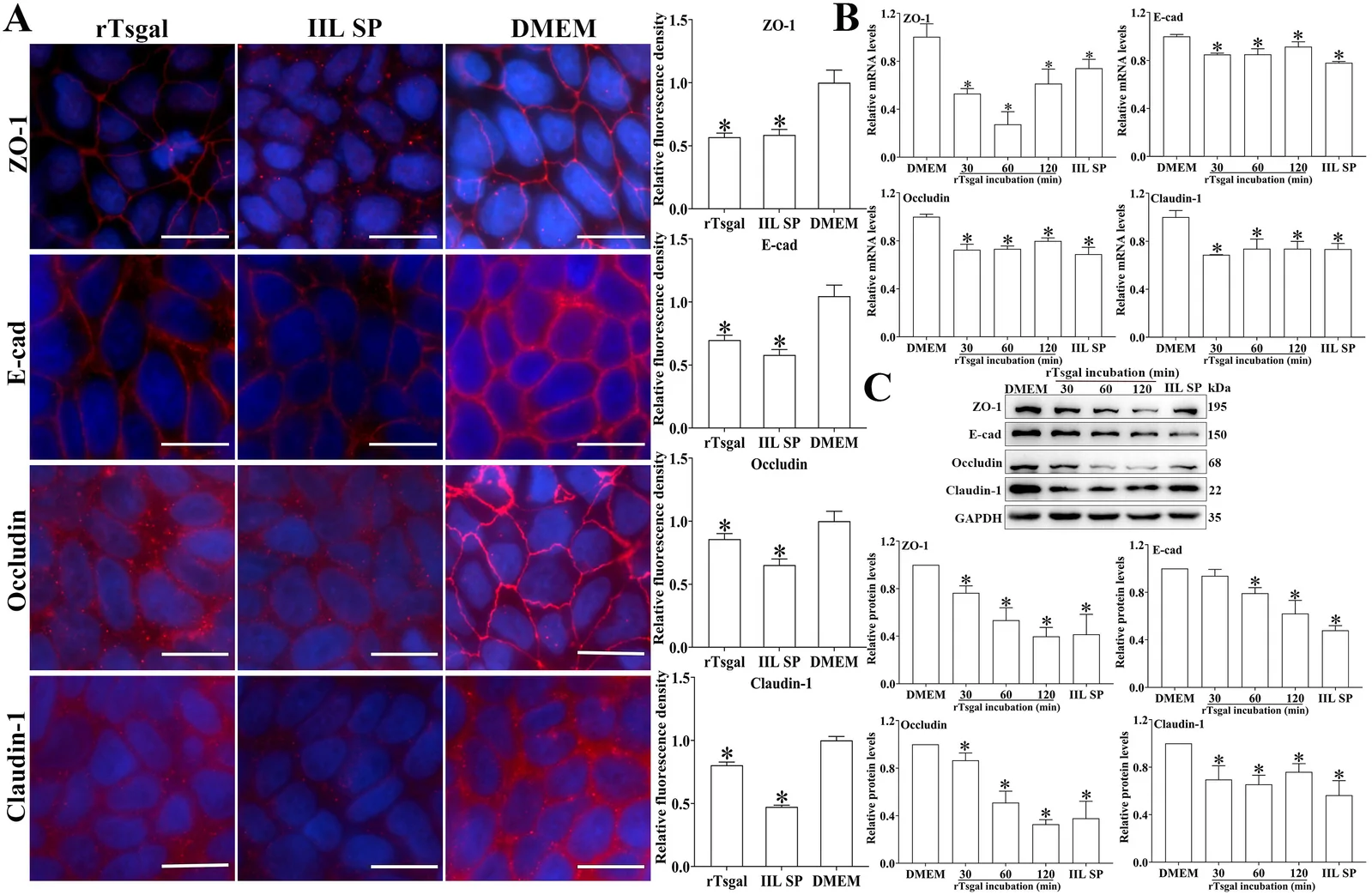
rTsgal decreased the TJs expression of Caco-2 monolayer. **A:** Immunofluorescence images of rTsgal-treated Caco-2 cells and stained for ZO-1, E-cad, Occludin and Claudin-1 (Red) and nuclei (DAPI/blue). Scale bar: 25 μm. **B:** qPCR assay of the TJs transcription level in Caco-2 monolayers treated with rTsgal. GAPDH was served as an internal reference. **C:** Western blot of TJs expression in Caco-2 monolayers after treatment with rTsgal. The relative protein levels in the DMEM group were used to normalize the TJs protein levels in other groups. Each assay had three replicates. Data were analyzed using one-way ANOVA, ^*^P < 0.05 compared to DMEM group.

The IFT revealed that the fluorescent intensity of TJs (ZO-1, E-cad, Occludin and Claudin-1) was significantly reduced in the rTsgal group in comparison to DMEM group (*F*_ZO-1_ = 41.248, *P* < 0.001; *F*_E-cad_ = 47.195, *P* < 0.001; *F*_Occludin_ = 26.241, *P* = 0.025; *F*_Claudin-1_ = 354.414, *P* < 0.001), they presented the linear localization, diffuse or discontinuous distribution among the cells (Fig 3A). The TJs mRNA levels were measured by qPCR. The qPCR data showed that compared with DMEM group, the TJs mRNA expression levels in rTsgal group were reduced significantly (*F*_ZO-1_ = 24.109, *P* < 0.001; *F*_E-cad_ = 24.335, *P* = 0.005; *F*_Occludin_ = 32.620, *P* < 0.001; *F*_Claudin-1_ = 15.406, *P* < 0.001) (Fig 3B). Western blotting displayed that in compared with the DMEM group, the TJs protein expression in rTsgal group were significantly down-regulated after incubation for 120 min (*F*_ZO-1_ = 20.359, *P* < 0.001; *F*_E-cad_ = 36.694, *P* < 0.001; *F*_Occludin_ = 53.757, *P* < 0.001; *F*_Claudin-1_ = 10.283, *P* < 0.008) (Fig 3C). These results demonstrated that rTsgal stimulation prominently downregulated the expression of ZO-1, E-cad, Occludin and Claudin-1, implicating that rTsgal disrupted Caco-2 monolayer integrity and intercellular junction stability.

### rTsgal up-regulated MUC13-ROCK2/MAPK expression

**Fig 4:**
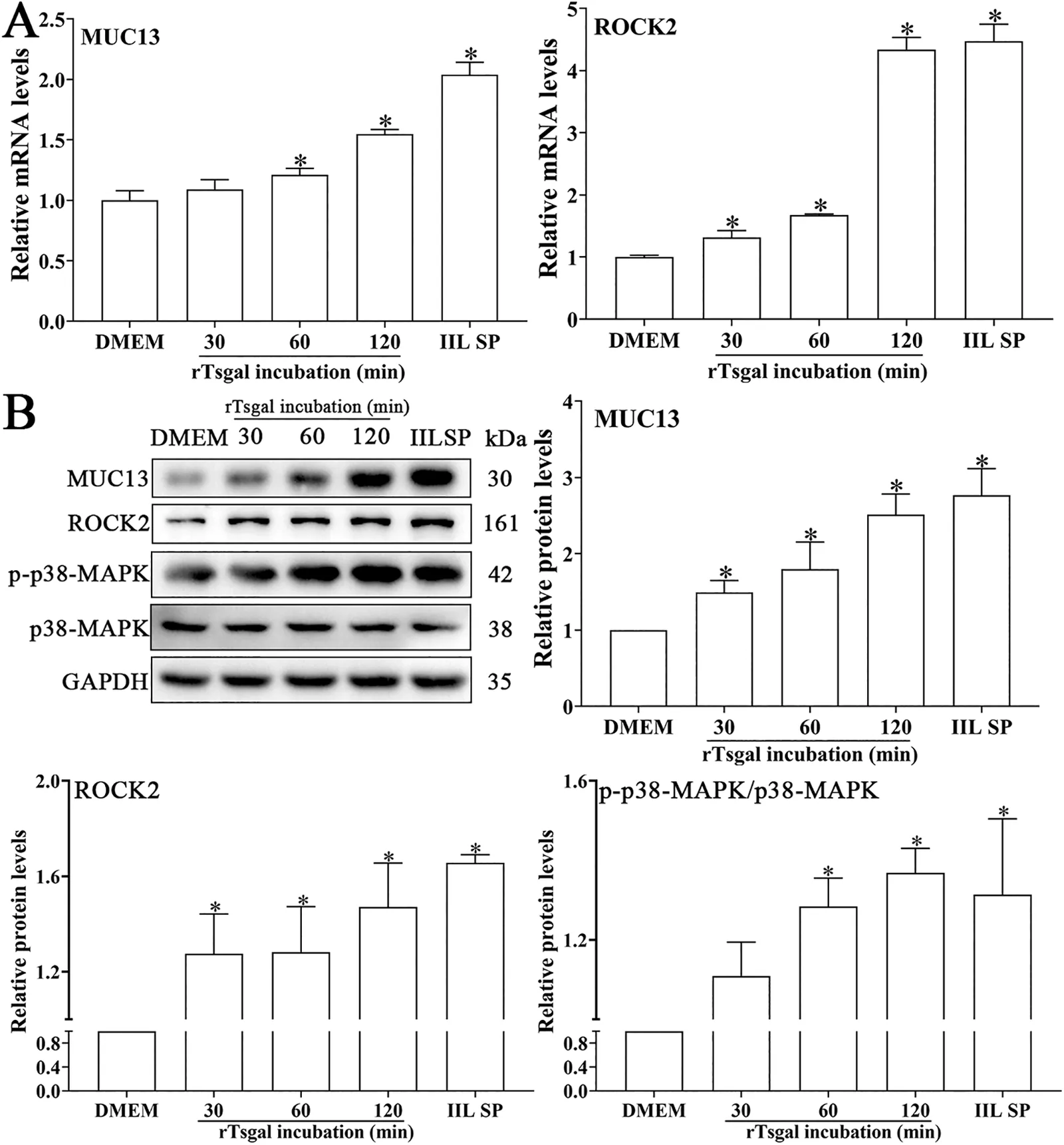
rTsgal activated MUC13-ROCK2/MAPK pathways. Caco-2 cells were treated with rTsgal for 30, 60 and 120 min. **A:** qPCR assay of transcription level of MUC13 and ROCK2, relative to DMEM group. GAPDH was served as an internal control. **B:** Western blot of expression of the MUC13, ROCK2 and p38-MAPK phosphorylation levels compared to the DMEM group. Data were presented as mean ± SD (n = 3), and analyzed using one-way ANOVA, ^*^P < 0.05 compared to the DMEM group.

To further assess whether the interaction of rTsgal and MUC13 activates MUC13-mediated ROCK2/MAPK pathways, we ascertained the expression levels of MUC13 and ROCK2/MAPK pathways in Caco-2 cells stimulated by rTsgal. qPCR revealed that rTsgal treatment for 120 min significantly increased the transcription level of MUC13 and ROCK2 compared with DMEM group (*F*_MUC13_ = 100.283, *F*_ROCK2_ = 343.796, *P* < 0.001) (Fig 4A). Western blot revealed that rTsgal treatment for 120 min distinctly elevated expression of MUC13, ROCK2 and p-p38-MAPK/p38-MAPK, compared with DMEM group (*F*_MUC13_ = 23.056, *P* < 0.001; *F*_ROCK2_ = 9.289, *P* = 0.002; *F*_p-p38-MAPK/p38-MAPK_ = 6.84, *P* = 0.001). The results confirmed that rTsgal activated the MUC13-ROCK2/MAPK signal pathways in Caco-2 cells (Fig 4B).

### MUC13 inhibitor suppressed the rTsgal-activated MUC13-ROCK2/MAPK pathways

**Fig 5:**
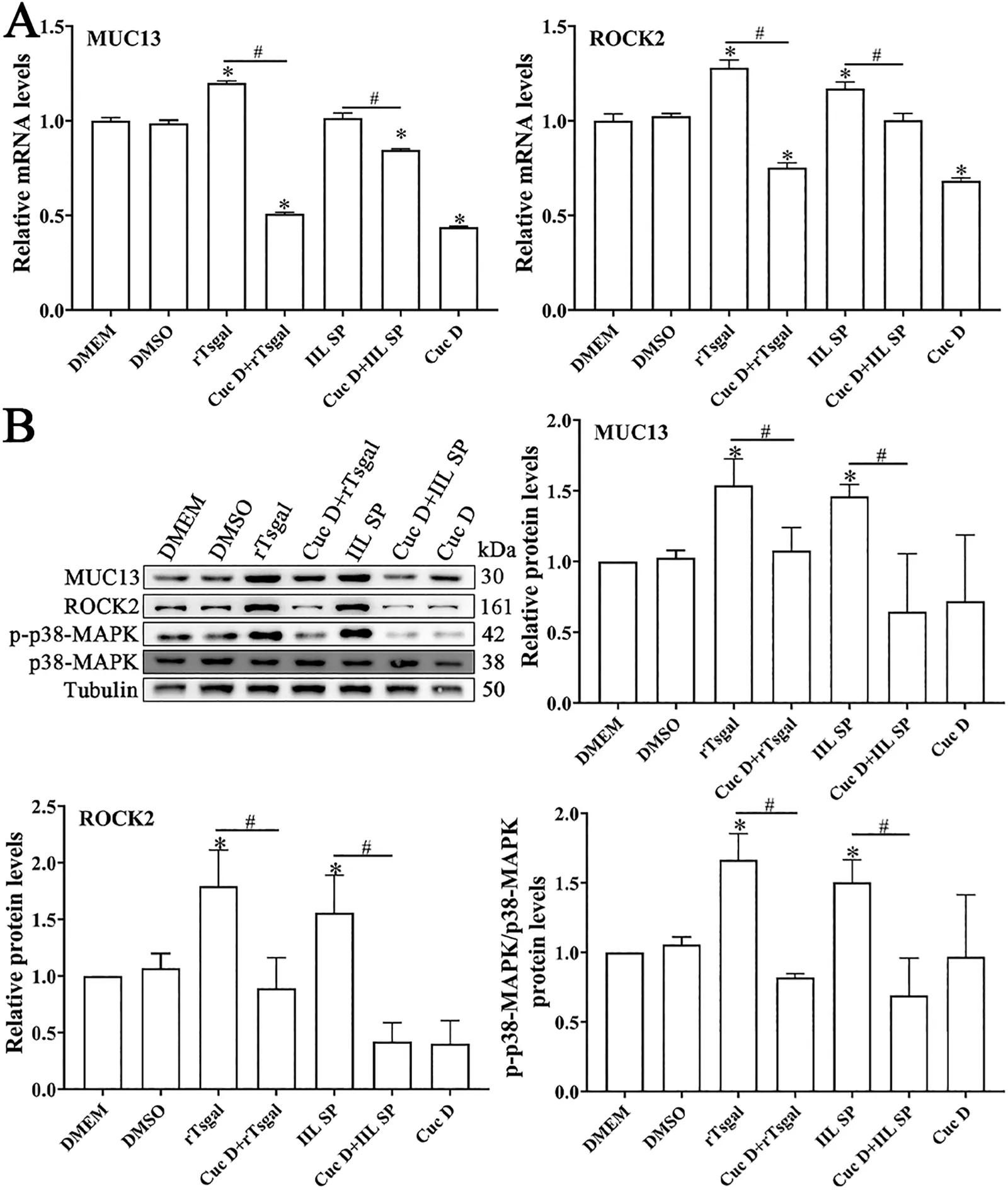
Cuc D inhibited the rTsgal-activated MUC13-ROCK2/MAPK pathways. Caco-2 cells were first pretreated with Cuc D for 24 h, and then incubated with rTsgal for 2 h. **A:** qPCR analysis of ROCK2 and MUC13 mRNA expression of Caco-2 cells normalized to GAPDH. **B:** Western blot analysis of MUC13, ROCK2 and p-p38-MAPK/p38-MAPK protein expression levels of Caco-2 cells. Data were presented as mean ± SD (n = 3) and analyzed using one-way ANOVA, ^*^P < 0.05 compared to DMEM group. Data were analyzed using Student’s t-test, ^#^*P* < 0.05 compared between the two groups.

The qPCR data demonstrated that rTsgal evidently upregulated the mRNA transcript levels of MUC13 and ROCK2, compared to DMEM group (*F*_MUC13_ = 1114.538, *F*_ROCK2_ = 146.982, *P* < 0.001). Conversely, in the Cuc D + rTsgal group, transcript levels of MUC13 and ROCK2 were significantly diminished, compared with the only rTsgal group (*t*_MUC13_ = 91.656, *t*_ROCK2_ = 19.143, *P* < 0.001) (Fig 5A). Western blotting analysis displayed that rTsgal stimulation markedly increased MUC13, ROCK2 and p-p38-MAPK expression, in comparison with the DMEM group (*F*_MUC13_ = 5.216, *P* = 0.022; *F*_ROCK2_ = 8.824, *P* = 0.004; *F*_p-p38-MAPK/P38-MAPK_ = 7.927, *P* = 0.002). Controversy, in Cuc D + rTsgal group, the expression levels of MUC13, ROCK2 and p-p38-MAPK were remarkably declined, compared with the only rTsgal group (*t*_MUC13_ = 3.22, *P* = 0.032; *t*_ROCK2_ = 3.378, *P* = 0.028; *t*_p-p38-MAPK/p38-MAPK_ = 7.695, *P* = 0.015) (Fig 5B). These findings revealed that rTsgal definitely activated MUC13-ROCK2/MAPK pathways, whereas MUC13 inhibitor Cuc D abolished rTsgal’s role for activating MUC13-ROCK2/MAPK pathways.

### MUC13 inhibitor relieved the rTsgal-destroyed Caco-2 monolayer barrier function

**Fig 6:**
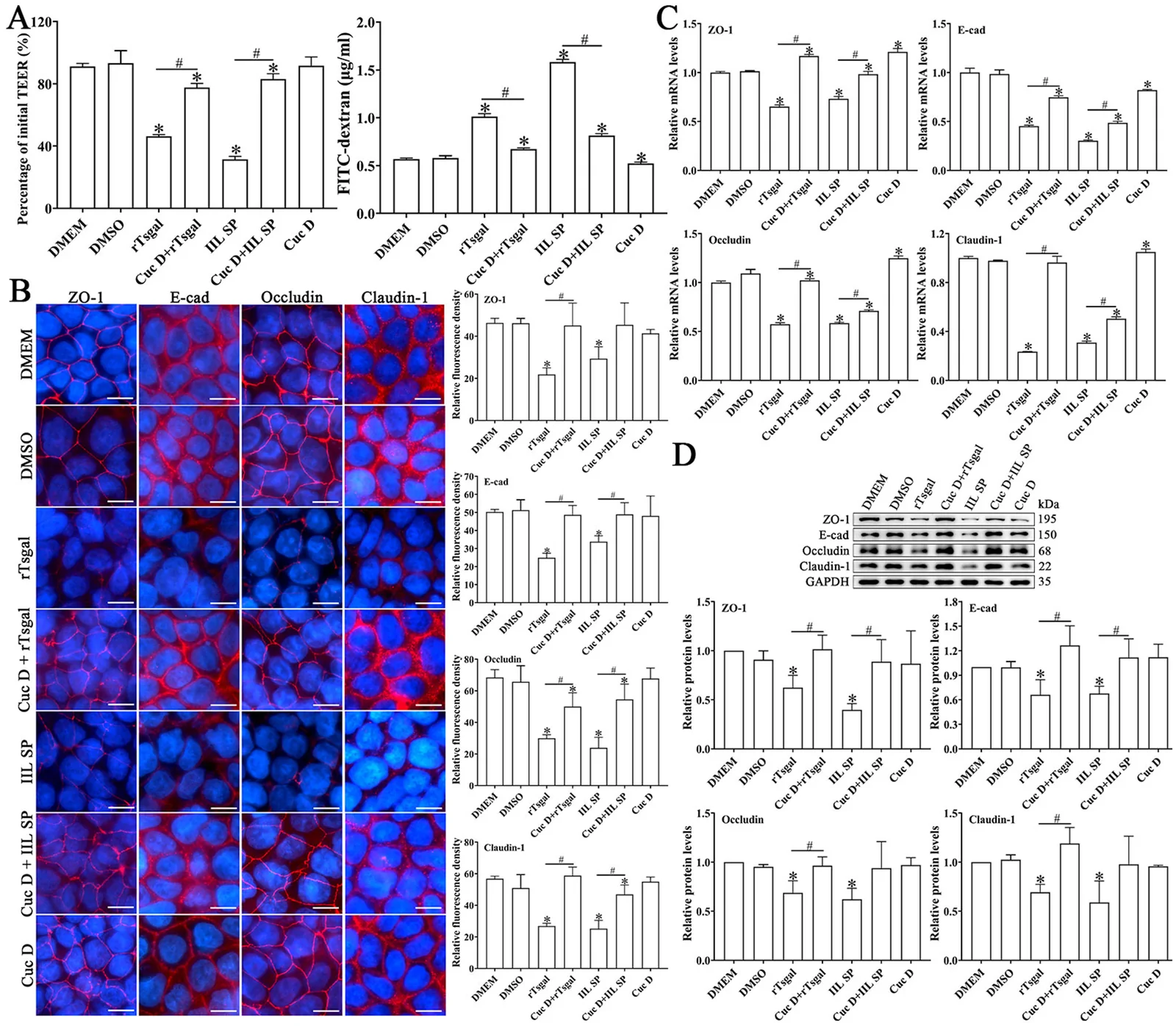
MUC13 inhibitor alleviated the rTsgal-disrupted intestinal epithelial barrier. **A:** Trans-epithelial electrical resistance (TEER) and FITC-dextran flux. **B:** IFT of TJs expression and distribution in Caco-2 monolayer incubated with Cuc D and rTsgal. Scale bars: 25 μm. **C:** qPCR assay of the TJs transcription in Caco-2 monolayers treated with Cuc D and rTsgal. GAPDH was served as an internal reference. **D:** Western blot of TJs expression in Caco-2 monolayers after incubation with Cuc D and rTsgal. Data were presented as mean ± SD (n = 3) and analyzed using one-way ANOVA, ^*^P < 0.05 compared to DMEM group. Data were analyzed using Student’s t-test, ^#^*P* < 0.05 compared between the two groups.

To asses MUC13’s contribution to rTsgal-mediated barrier damage, Caco-2 monolayers were pretreated with 1 μM Cuc D prior to rTsgal exposure. The TEER measurement demonstrated that pretreatment with the MUC13 inhibitor Cuc D prior to rTsgal exposure, substantially attenuated the rTsgal-induced TEER decrease, relative to the rTsgal alone group (*t* = 19.036, *P* < 0.001). The flux of FD-4 was significantly diminished in the Cuc D + rTsgal group relative to rTsgal group (*t* = 17.668, *P* < 0.001) (Fig 6A). The IFT analysis indica*t*ed that rTsgal alone markedly diminished contents of TJs (ZO-1, E-cad, Occludin and Claudin-1) in Caco-2 cells compared with the DMEM group (*F*_ZO-1_ = 7.276, *P* = 0.001; *F*_E-cad_ = 9.007, *F*_Occludin_ = 17.671, *F*_Claudin-1_ = 22.97, *P* < 0.001). However, pretreatment with Cuc D significantly restored and fortified the rTsgal-reduced TJs compared with only rTsgal group (*t*_ZO-1_ = 3.629, *P* = 0.022; *t*_E-cad_ = 7.14, *P* = 0.002; *t*_Occludin_ = 3.829, *P* = 0.019; *t*_Claudin-1_ = 9.734, *P* < 0.001) (Fig 6B). Pretrea*t*men*t* of Caco-2 cells with Cuc D drastically restored and elevated the rTsgal-reduced mRNA transcript levels of TJs, according to qPCR analysis (*t*_ZO-1_ = 36.46, *t*_E-cad_ = 26.019, *t*_Occludin_ = 33.824, *t*_Claudin-1_ = 25.003, *P* < 0.001) (Fig 6C). Western blo*t* indicated that Cuc D pretreatment obviously increased TJs expression relative to rTsgal group (*t*_ZO-1_ = 3.260, *P* = 0.024; *t*_E-cad_ = 3.459, *P* = 0.026; *t*_Occludin_ = 3.153, *P* = 0.034; *t*_Claudin-1_ = 4.735, *P* = 0.039) (Fig 6D). All of these findings certified that rTsgal-disrupted Caco-2 monolayer integrity was mitigated by MUC13 receptor inhibition, and further suggested that MUC13 pathway participated in the rTsgal damaging to intestinal epithelial barrier integrity.

### Knockdown MUC13 gene

After Caco-2 cells were transfected with 50 nM siRNA for 48 h, the MUC13 transcript levels in the siMUC13#1 and siMUC13#2 group were reduced to 0.77 (*F* = 36.438, *P* = 0.001) and 0.6 folds (*F* = 36.438, *P* < 0.001) of DMEM group, separately (S5A Fig), and there was no statistical distinction between siNC group and DMEM group (*P* = 0.704). The MUC13 protein level in siMUC13#1 and siMUC13#2 was reduced to 0.88 (*F* = 24.974, *P* = 0.04) and 0.65 folds (*F* = 24.974, *P* < 0.001) of DMEM group, separately. And there was no statistical distinction between siNC group and DMEM group (*P* = 0.474) (S5B Fig). The findings indicated that both siMUC13#1 and siMUC13#2 have good inhibitory effects on MUC13 expression in Caco-2 cells, and siMUC13#2 has a better inhibitory effect than siMUC13#1 (*P* = 0.007). Therefore, siMUC13#2 is used for subsequent experiments. The MUC13 transcript levels in Caco-2 transfected with 25, 50 and 100 nM siMUC13 for 2 d were reduced to 0.86 (*F* = 41.243, *P* = 0.015), 0.72 (*F* = 41.243, *P* < 0.001) and 0.54 folds (*F* = 41.243, *P* < 0.001) of DMEM group, separately (S5C Fig). The MUC13 protein expression levels in above 3 dose groups were reduced to 0.9 (*F* = 19.163, *P* = 0.3), 0.71 (*F* = 19.163, *P* = 0.008) and 0.37 folds (*F* = 19.163, *P* < 0.001) of DMEM group. The results indicated that 100 nM siMUC13 has a better inhibitory effect on MUC13 expression (S5D Fig). The MUC13 mRNA levels in Caco-2 cells transfected with 100 nM siMUC13 for 1, 2 and 3 d were reduced to 0.92 (*F* = 0.385, *P* = 0.548), 0.51 (*F* = 79.248, *P* < 0.001) and 1-fold (*F* = 8.217, *P* = 0.263) of DMEM group (S5E Fig). The MUC13 protein levels in Caco-2 transfected with 100 nM siMUC13 for 1, 2 and 3 d were respectively reduced to 0.73 folds (*F* = 16.807, *P* = 0.003), 0.41 folds (*F* = 51.273, *P* < 0.001) and 0.77 folds (*F* = 39.647, *P* < 0.001) of DMEM group (S5F Fig). Therefore, transfection of Caco-2 cells with 100 nM siMUC13#2 followed by a 48 h incubation was the optimal condition for efficiently silencing MUC13, achieving consistent 40–60% of MUC13 knockdown efficiency at both mRNA and protein levels, and these conditions were used in all subsequent experiments.

### MUC13 knockdown relieved the rTsgal-destroyed Caco-2 monolayer barrier function

**Fig 7:**
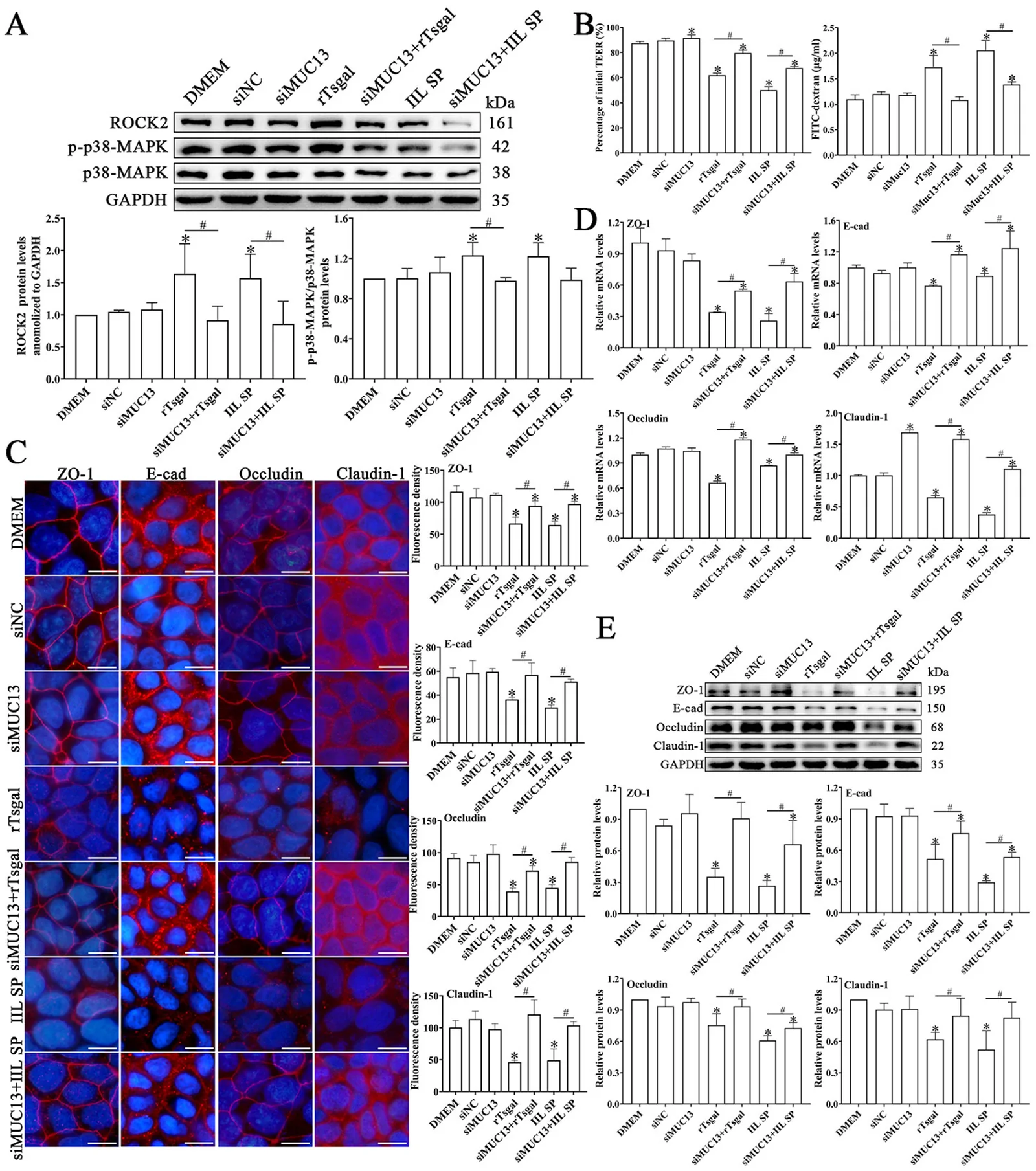
Caco-2 cell MUC13 knockdown abolished and alleviated the rTsgal-disrupted monolayer integrity. **A:** Western blotting of expression levels of ROCK2 and p-p38-MAPK/p38-MAPK. **B:** Trans-epithelial electrical resistance (TEER) and FITC-dextran flux of FD-4 through Caco-2 monolayer. **C:** Immunofluorescence images of the rTsgal and siMUC13 + rTsgal group of Caco-2 monolayer and stained for ZO-1, E-cad, Occludin and Claudin-1 (Red) and nuclei (DAPI/blue). Scale bars: 25 μm. **D:** The mRNA levels of TJs (ZO-1, E-cad, Occludin and Claudin-1) in rTsgal group and siMUC13 + rTsgal group. GAPDH was served as an internal control. **E:** Western blotting of expression levels of TJs (ZO-1, E-cad, Occludin and Claudin-1) in the rTsgal and siMUC13 + rTsgal. The relative protein levels in the DMEM group were used to normalize protein levels in other groups. Each assay had a triplicate. Data were presented as mean ± SD (n = 3) and analyzed using one-way ANOVA, ^*^P < 0.05 compared to DMEM group, ^#^*P* < 0.05 compared between the two groups.

To ascertain the MUC13’s roles in rTsgal-actived ROCK2/MAPK pathways and rTsgal-disrupted intestinal epithelial barrier, the MUC13-knockdown Caco-2 cells were used. Western blot exhibited that the rTsgal treatment upregulated expression levels of ROCK2 and p-p38-MAPK compared with the DMEM group. Additionally, following rTsgal treatment, MUC13-knockdown Caco-2 cells’ ROCK2 and p-p38-MAPK expression levels were considerably declined relative to rTsgal group (normal Caco-2 cells) (ROCK2: *F* = 3.827, *P* = 0.014; p-p38-MAPK/p38-MAPK: *F* = 3.219, *P* = 0.012) (Fig 7A). TEER assay revealed that rTsgal significantly decreased TEER (*F* = 188.38, *P* < 0.001) and increased FITC-Dextran permeability (*F* = 26.596, *P* < 0.001), in comparison to DMEM group. However, knockdown MUC13 clearly abolished and increased the rTsgal-decreased TEER (*P* < 0.001), and decreased the rTsgal-increased FITC-Dextran permeability (*P* < 0.001) (Fig 7B). Moreover, IFT, qPCR, and Western blotting were used to assess the MUC13 role in rTsgal-reducing the expression of TJs (ZO-1, E-cad, Occludin and Claudin-1). The TJs level in the rTsgal-treated Caco-2 cells was clearly lower than the DMEM group, based on the IFT images. MUC13 knockdown effectively prevented rTsgal from reducing TJs expression levels (ZO-1: *F* = 19.153, *P* = 0.001; E-cad: *F* = 10.043, *P* = 0.026; Occludin: *F* = 22.151, *P* < 0.001; Claudin-1: *F* = 15.863, *P* < 0.001) (Fig 7C). qPCR results exhibited that mRNA level of ZO-1, E-cad, Occludin and Claudin-1 in siMUC13 + rTsgal group was significant higher than rTsgal group (ZO-1: *F* = 37.444, *P* = 0.008; E-cad: *F* = 10.032, *P* < 0.001; Occludin: *F* = 176.216, *P* < 0.001; Claudin-1: *F* = 400.142, *P* < 0.001) (Fig 7D). Western blot revealed that in comparison to the DMEM group, rTsgal markedly decreased TJs expression (*F*_ZO-1_ = 15.477, *P* = 0.02; *F*_E-cad_ = 28.195, *P* < 0.001; *F*_Occludin_ = 15.657, *P* < 0.001; *F*_Claucin-1_ = 5.754, *P* = 0.002). Furthermore, pre-knockdown MUC13 restored and increased the rTsgal-reduced TJs expression level (ZO-1: *F* = 15.477, *P* < 0.001; E-cad: *F* = 28.195, *P* = 0.004; Occludin: *F* = 15.657, *P* = 0.005; Claudin-1: *F* = 5.754, *P* = 0.002) (Fig 7E). Overall, these data suggested that rTsgal obviously activated ROCK2/MAPK pathways to disrupt intestinal epithelial integrity through binding and interacting with MUC13 receptor. This mechanism revealed the pivotal involvement of MUC13 in rTsgal-mediated intestinal barrier dysfunction.

### Inhibitors of ROCK2 and p38-MAPK restored and alleviated the rTsgal-destroyed gut epithelial integrity

**Fig 8:**
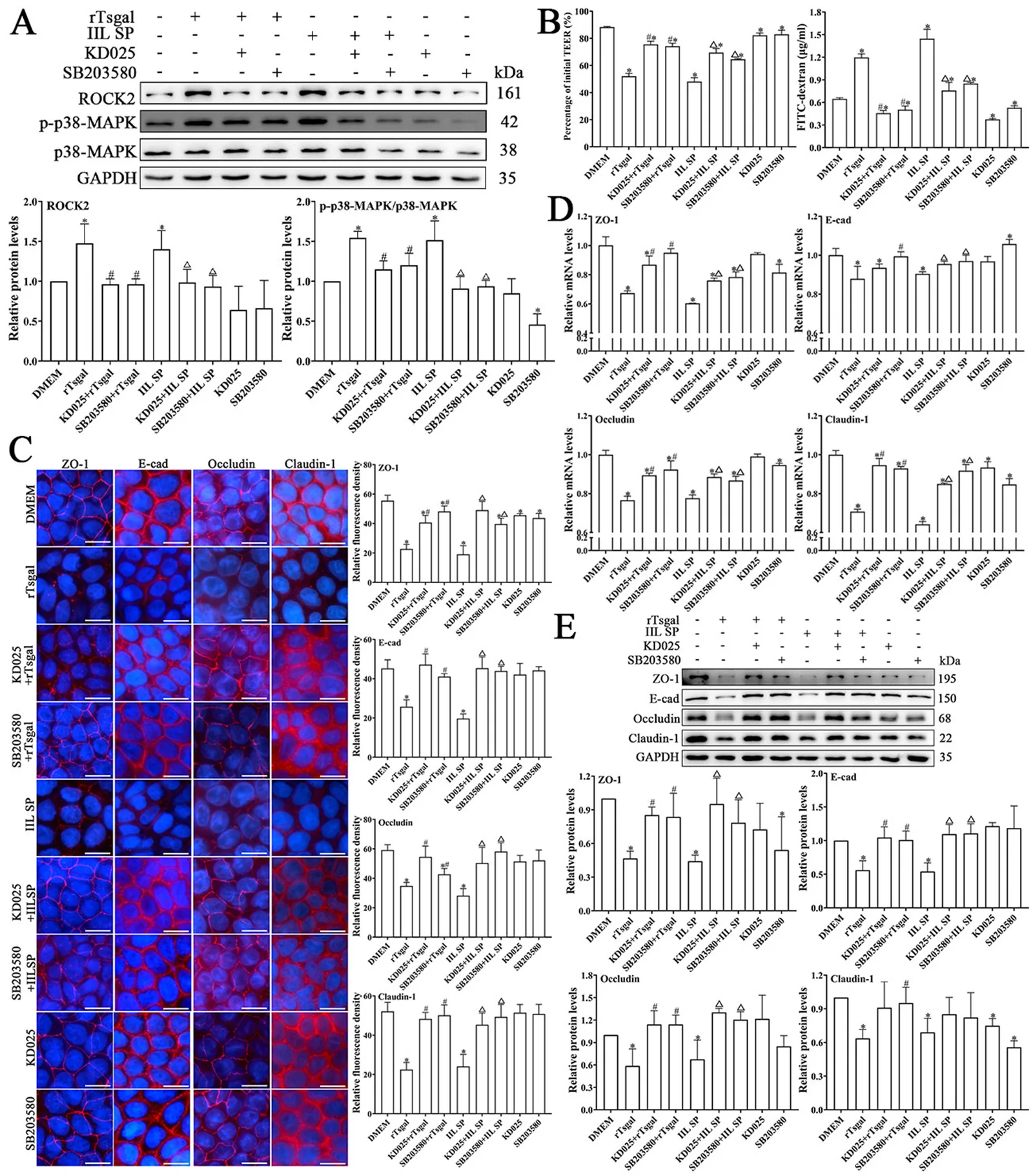
Inhibition of ROCK2/MAPK alleviated the rTsgal-damaged Caco-2 monolayer barrier integrity. **A:** Caco-2 cells were first pretreated with 2 μM KD025 and 1 μM SB203580 for 1 h, and then incubated with rTsgal for 2 h. Expression levels of ROCK2 and p-p38-MAPK/p38-MAPK were analyzed with Western blot. **B:** Trans-epithelial electrical resistance (TEER) and FITC-dextran flux. **C:** Immunofluorescence staining of intestinal epithelial TJs proteins (ZO-1, E-cad, Occludin and Claudin-1). Scale bars: 25 μm. **D:** qPCR assay of the TJs transcription in Caco-2 monolayer treated with inhibitors and rTsgal. GAPDH was served as an internal control. **E:** Western blot analysis of expression levels of ZO-1, E-cad, Occludin and Claudin-1 in the Caco-2 monolayer treated with inhibitors and rTsgal. The relative protein levels in the DMEM group were used to normalize protein levels in other groups. Each assay had three replicates. Data were analyzed using one-way ANOVA, ^*^P < 0.05 compared to the DMEM group. Data were analyzed using Student’s t-test, ^#^*P* < 0.05 compared to the rTsgal alone group, ^△^*P* < 0.05 compared to the IIL SP group.

To examine the function of ROCK2/MAPK pathways in rTsgal-disrupted intestinal epithelial integrity, ROCK2 inhibitor (KD025) and p38-MAPK inhibitor (SB203580) were used in this study. Western blot revealed that when Caco-2 monolayer was pretreated by either KD025 or SB203580, expression level of ROCK2 and p38-MAPK in KD025 + rTsgal and SB203580 + rTsgal groups were evidently inhibited and decreased compared to the rTsgal alone group (ROCK2: *t*_KD025_ = 3.78, *P* = 0.019; *t*_SB203580_ = 3.52, *P* = 0.024; p-p38-MAPK/p38-MAPK: *t*_KD025_ = 5.099, *P* = 0.007; *t*_SB203580_ = 3.492, *P* = 0.025) (Fig 8A). Additionally, the rTsgal-damaged gut epithelial barrier was abolished and relieved by pretreating Caco-2 cells with ROCK2 inhibitor (KD025) or p38-MAPK inhibitor (SB203580), as evidenced by the increased TEER (*t*_KD025_ = 12.673, *t*_SB203580_ = 12.576, *P* < 0.001) and decreased FD-4 permeability (*t*_KD025_ = 21.976, *t*_SB203580_ = 17.822, *P* < 0.001) in KD025 + rTsgal and SB203580 + rTsgal groups (Fig 8B). The IFT analysis confirmed that the fluorescent density of ZO-1, E-cad, Occludin and Claudin-1 was recovered in KD025 + rTsgal and SB203580 + rTsgal group compared with the only rTsgal group (ZO-1: *t*_KD025_ = 5.371, *P* = 0.006; *t*_SB203580_ = 9.03, *P* < 0.001; E-cad: *t*_KD025_ = 5.74, *P* = 0.005; *t*_SB203580_ = 6.97, *P* = 0.002; Occludin: *t*_KD025_ = 4.402, *P* = 0.012; *t*_SB203580_ = 2.958, *P* = 0.042; Claudin-1: *t*_KD025_ = 9.128, *P* < 0.001; *t*_SB203580_ = 7.496, *P* = 0.002) (Fig 8C). qPCR exhibited that after rTsgal stimulation, the TJs mRNA levels were distinctly increased in two inhibitors-pretreated groups (ZO-1: *t*_KD025_ = 12.433, *P* < 0.001; *t*_SB203580_ = 15.15, *P* < 0.001; E-cad: *t*_SB203580_ = 2.991, *P* = 0.044; Occludin: *t*_KD025_ = 9.606, *P* < 0.001; *t*_SB203580_ = 5.569, *P* = 0.005; Claudin-1: *t*_KD025_ = 11.231, *P* < 0.001; *t*_SB203580_ = 24.525, *P* < 0.001). However, compared with rTsgal alone group, the transcription level of E-cad was not obviously increased in KD025+rTsgal group (*t* = 1.444, *P* = 0.222) (Fig 8D). Subsequent Wes*t*ern blo*t*ting revealed that two inhibitors (KD025 and SB203580) eliminated and reversed the rTsgal-decreased TJs levels in KD025 + rTsgal and SB203580 + rTsgal groups (ZO-1: *t*_KD025_ = 6.856, *P* = 0.002; *t*_SB203580_ = 2.915, *P* = 0.043; E-cad: *t*_KD025_ = 5.333, *P* = 0.006; *t*_SB203580_ = 7.361, *P* = 0.002; Occludin: *t*_KD025_ = 3.279, *P* = 0.031; *t*_SB203580_ = 3.702, *P* = 0.021; Claudin-1: *t*_SB203580_ = 5.488, *P* = 0.005). However, compared with rTsgal alone group, the expression level of Claudin-1 was not increased in KD025 + rTsgal group (*t* = 1.918, *P* = 0.128) (Fig 8E). These results showed that rTsgal binding to the MUC13 receptor in Caco-2 cells triggered the ROCK2/MAPK pa*t*hway, decreased the expression of the TJs protein, and destroyed the integrity and barrier function of gut epithelium, all of which facilitated *T. spiralis* IIL to intrude into the host gut mucosa.

### Inhibition of MUC13 and ROCK2/MAPK prevented larva invasion of Caco-2 monolayer

**Fig 9:**
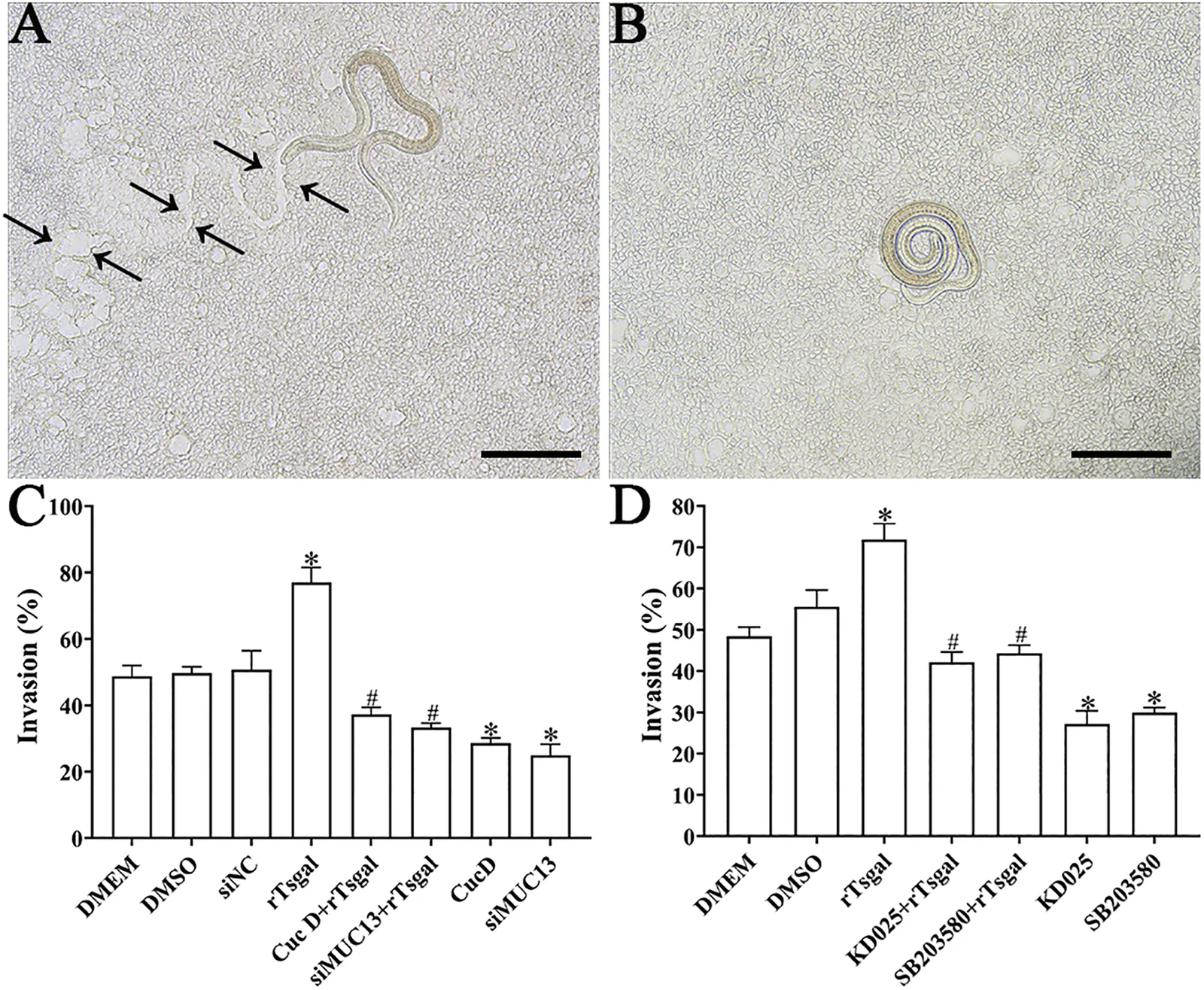
Inhibition of MUC13 and ROCK2/MAPK suppressed larval invasion of Caco-2 monolayer. **A:** The invaded larva was migrated within the Caco-2 monolayer. **B:** The non-invaded larva was spirally coiled on the monolayer and suspended in the medium. **C:** MUC13 inhibition or knockdown abolished and reduced rTsgal-increased larval invasion. **D:** Pretreatment of Caco-2 monolayer with ROCK2 inhibitor (KD025) or p38-MAPK inhibitor (SB203580) obviously inhibited the larval invasion of Caco-2 monolayers. Each assay had three replicates. Data were analyzed using Chi-square test, ^*^P < 0.05 compared to the DMEM group. ^#^*P* < 0.05 compared to only rTsgal group. Scale bars: 200 μm.

The *in vitro* invasive assay revealed that the penetrated larvae produced visible migratory tracks through Caco-2 monolayers (Fig 9A, black arrow), whereas non-penetrated larvae coiled spirally on cellular surface (Fig 9B). Larval invasion rate of the rTsgal group was 76.98%, which was much greater than the DMEM group’s 48.79%, rTsgal dramatically increased larval incursion when applied to Caco-2 monolayer (*χ²* = 5.956, *P* = 0.015). Moreover, this facilitative effect of rTsgal was substantially abolished when Caco-2 cells were pretreated with MUC13 inhibitor (Cuc D) or siMUC13 prior to rTsgal treatment (*χ²*_Cuc D +rTsgal_ = 16.568, *P* < 0.001; *χ²*_siMUC13+rTsgal_ = 19.165, *P* < 0.001), compared with the rTsgal alone group. Furthermore, when the IIL was administered to the Caco-2 monolayer and co-cultured for 2 h following the initial pretreatment with Cuc D or siMUC13, the larval invasion was markedly inhibited (*χ²*_Cuc D_ = 4.527, *P* = 0.033; *χ²*_siMUC13_ = 6.211, *P* = 0.013). Larva invasion of Cuc D and siMUC13 groups had only 29.00 and 25.01%, respectively (Fig 9C). Furthermore, the larval invasion was clearly decreased when Caco-2 cells were pretreated by ROCK2 inhibitor (KD025) or MAPK inhibitor (SB203580) and subsequently incubated with rTsgal, with an invasion reduction of 39.73% and 43.64%, respectively (*χ²*_KD025+rTsgal_ = 9.245, *P* = 0.002; *χ²*_SB203580+rTsgal_ = 8.171, *P* = 0.004), compared to the rTsgal alone group. Additionally, larval invasion of KD025 group (27.17%) or SB203580 group (29.93%) were lower than DMEM group (48.61%) (*χ²*_KD025_ = 4.719, *P* = 0.03; *χ²*_SB203580_ = 3.87, *P* = 0.049) (Fig 9D).The results showed that inhibition or knockdown of MUC13 receptor, and inhibition of ROCK2/MAPK pathway significantly impeded and reduced the IIL invasion of Caco-2 cell, and suggested that Tsgal played an important role in binding to MUC13 receptor and activating ROCK2/MAPK pathways, destroying gut epithelium integrity and barrier function, and finally mediating larva invasion.

## Discussions

Numerous intestinal disorders, such as bacterial and parasite infections, have been linked to the gut epithelial barrier dysfunction [54]. *Giardia* trophozoite ESP affect localization of gut apical junctional complex (AJC) proteins and recombinant *Giardia* cysteine proteases cleave or re-localize the AJC proteins (Claudin-1 and -4, Occludin, JAM-1, β-catenin and E-cadherin) of the IEC [55]. A serine protease from *T. spiralis* interacts with RACK1 receptor to disrupted gut barrier via ERK1/2 signaling [34]. Aspartic protease 2 from *T. spiralis* excretion/secretion products hydrolyzes tight junctions of intestinal epithelial cells [56]. In our previous study, a *T. spiralis* galectin interacted with TLR-4 receptor and activated NF-κB signaling, modulated intestinal inflammation and mediated larval invasion [23]. However, just a part of larval invasion was hindered when anti-rTsgal antibodies and TLR4 inhibitor (TAK-242) were utilized in the larval invasion assay, indicating that Tsgal mediated larval invasion through various mechanisms. These findings collectively supported the hypothesis that *T. spiralis* invasion gut mucosa is mediated by a coordinated and partially redundant effector network rather than a single protein molecule. Proteases might directly degrade the junctional proteins; lectins likely promote larval adhesion to gut epithelia and activate gut epithelial receptors and signaling pathways. Together, these *T. spiralis* proteins result in both structural destruction of apical junctional complex and rewiring of signaling responses. In this research, we investigated whether rTsgal disrupts intestinal barrier and facilitates *T. spiralis* larval invasion of gut epithelium, and further identifies the target molecules and mechanisms involved in the larval invasion. However, the synergistic effect of different *T. spiralis* proteins during the invasion process of host gut epithelium needs to be further investigated.

Proteins rarely function as a single substance, they usually as team players in a dynamic interaction network. Accumulating data demonstrate that associations between proteins play pivotal roles across diverse physiological pathways [57]. GST pull-down assay, Co-IP and LC-MS/MS are the conventional approaches of identifying or confirming the occurrence of protein-protein interaction, these techniques have been widely used to ascertain interaction of parasite and host [58,59]. In the present work, we focused on the GST pull-down coupled with LC-MS/MS as effective means to discover and verify the direct interactions between rTsgal and its potential binding partners. The results showed that MUC13 is one of the potential ligands of rTsgal. rTsgal is known as a tandem lectin protein containing two CRDs [18]. Mucins are highly glycosylated proteins and contain polylactosamine structures, and previous studies have shown that mucins are the ligand of galectin [60]. Molecular docking is a widely applied method in the field of structural molecular biology, which can supply useful information about protein–protein complex interactions [61]. In this study, rTsgal docked with MUC13 receptor and showed a strong affinity. IFT and Co-IP results further revealed that there were a binding and interaction between rTsgal and MUC13, and that rTsgal and MUC13 was colocalized in the cytomembrane. MUC13 is a transmembrane glycoprotein with high molecular weight; it protects and lubricates mucosal epithelium. It is typically expressed in gastrointestinal epithelial cells. Bacterial and parasitic infections strongly activate the cell surface mucin MUC13 [39,62]. It is composed of three domains that resemble epithelial growth factor (EGF), a cytoplasmic tail with potential phosphorylation sites, and a glycosylated extracellular domain that includes a sea urchin sperm protein enterokinase and agrin (SEA) domain. Diverse and intricate O-linked oligosaccharides are expressed by the MUC13 extracellular domain [63]. A common mucin trait is diverse oligosaccharide structures in the glycocalyx, which pathogens evolved to bind. MUC13 facilitates direct interaction between invasive pathogens and the host, and participates in regulating intestinal barrier. Intestinal epithelium barrier function is enhanced via destroying MUC13 O-linked oligosaccharides domain in *Bifidobacterium bifidum* strains [64]. MUC13 functions as a pathogen-binding decoy and interacts with SiiE adhesin of *Salmonella Typhimurium.* Additionally, MUC13 decreases intestinal barrier disruption and epithelial cell death via increasing NF-κB signaling during infection [65]. By altering the JAK1-STAT3, SNAI1-ZEB1, and ROCK2-MAPK signaling, IL-22-enhanced MUC13 expression affected intestinal barrier [66]. However, The direct rTsgal-MUC13 interaction requires further validation by using surface plasmon resonance (SPR) or fluorescence resonance energy transfer (FRET) [38]. Moreover, binding site of Tsgal binding with MUC13 remains to be determined in future work to enable mechanism-based therapeutic targeting. Furthermore, our mutational docking analysis showed that mutations of rTsgal CRD region (His60, Arg70, and Lys87) reduced the predicted binding affinity with MUC13, provided additional structural evidence supporting the rTsgal CRD region contribution to the rTsgal–MUC13 interaction interface. Although these findings support the interaction between rTsgal and MUC13, quantitative approaches such as SPR, FRET, or competitive binding assays will confirm the binding affinity and kinetics of this interaction. Further application of combined structural, biochemical and functional approaches will elucidate the precise molecular mechanism by which rTsgal recognizes MUC13 and disrupts intestinal epithelial barrier.

The TJs constitute the essential intercellular complexes regulating paracellular permeability, and sustaining the gut epithelial barrier function [67]. As the disruption of gut barrier is a critical driver of *Trichinella* invasion [68], we evaluate whether rTsgal disrupts gut barrier integrity. The TJ complex comprises zona occluden proteins, E-cad, Occludin, and Claudins, contributes to epithelial barrier maintenance [69,70]. Consequently, we quantified mRNA and protein expression levels of ZO-1, E-cad, Occludin and Claudin-1 by using IFT, qPCR and Western blot. The results showed that transcription and expression of ZO-1, E-cad, Occludin and Claudin-1 in the rTsgal-incubated Caco-2 monolayer were significantly decreased, indicating that Tsgal reduced and declined the TJs expression levels.

It has been reported that MUC13-activated JAK1/STAT3, ROCK2/MAPK and protein kinase C (PKC) impact gut epithelial barrier [66,71]. In this study, we also assessed whether the interaction of rTsgal with MUC13 activates the MUC13-mediated downstream signaling, and the MUC13-related ROCK2/MAPK pathways were assayed. Interestingly, expression level of MUC13 and ROCK2/MAPK in rTsgal-stimulated Caco-2 monolayer was significantly increased. The result implied that rTsgal might bind and up-regulate MUC13, activated MUC13-related ROCK2/MAPK pathways, decreased TJs expression and disrupted gut epithelial integrity, and finally promoted larva invasion [11,34]. Additionally, when MUC13 receptor inhibitor Cuc D was used or MUC13 was knockdown by specific siRNA, the rTsgal-triggered ROCK2/MAPK activation and TJs disruption was significantly inhibited and abrogated, suggesting that gut MUC13 receptor plays an important role in rTsgal disrupting gut epithelial integrity.

ROCK2 functions as a principal modulator of actin cytoskeleton and cell polarity [72]. The MAPK signaling governs cellular growth, activation, mitotic division, stress adaptation, inflammatory response, and diverse fundamental physiological process [73]. Notably, MAPK signaling also is the central pathway in intestinal epithelial barrier compromise [74]. Our experimental observations revealed that in Caco-2 monolayer pretreated with MUC13 inhibitor Cuc D or with MUC13 knockdown, the ROCK2/MAPK pathway was also inhibited. To further confirm whether rTsgal-damaged gut epithelium integrity is related with ROCK2/MAPK pathway activation, ROCK2 inhibitor (KD025) and MAPK inhibitor (SB203580) were also used. The findings demonstrated that ROCK2 and p-p38-MAPK expression levels in Caco-2 monolayers pretreated with either KD025 or SB203580 were significantly lower than the rTsgal alone group. Additionally, the rTsgal-damaged gut epithelial barrier was also relieved. The results implied that activation of MUC13/ROCK2/MAPK pathway play a key role in rTsgal-destructed gut epithelial barrier.

Furthermore, the interaction between Tsgal and MUC13 may represent an upstream trigger that activates multiple downstream signaling cascades, including the ROCK2 and MAPK signaling. ROCK2 is a key regulator of actin cytoskeleton dynamics, including actin polymerization, stress fiber formation and epithelial junctional tension, which are closely related to the formation and arrangement of tight junction proteins [75]. Moreover, it has been reported that MAPK signaling pathways (p38-MAPK, ERK1/2 and JNK) regulated tight junction proteins and cytoskeletal dynamics via phosphorylation-dependent mechanisms, thereby further exacerbating epithelial barrier dysfunction [76]. Importantly, ROCK2-mediated cytoskeletal contraction and MAPK-dependent phosphorylation may act synergistically to destabilize gut epithelial barrier. Further investigation on the downstream signaling components, including phospho-specific detection of MAPK pathway proteins and visualization of actin remodelling, would provide a deeper mechanism of rTsgal mediating larval invasion.

The first important stage of *T. spiralis* infection is the IIL penetration of gut mucosa [77]. Previous studies showed that three pivotal indispensable requirements for the *in vitro* IIL invasion are the agarose physical support, larvae bile activation and epithelial cell monolayers [78]. Findings from our *in vitro* invasion assay demonstrated that rTsgal definitely helped the larval penetration into Caco-2 monolayer, whereas inhibition of MUC13, ROCK2 and MAPK substantially attenuated this invasive effect. However, Cuc D, KD025 and SB203580 are not specific inhibitor; they also can modulate other cellular functions. The ROCK family comprises two isoforms (ROCK1 and ROCK2), sharing 65% acid sequences homology overall, with their catalytic kinase domains displaying notably elevated conservation at 92% sequence identity. KD025 is widely used as a ROCK2-selective inhibitor, and has a selectivity of 75 − 100 fold for ROCK2 over ROCK1. Previous studies revealed that KD025 also inhibited ROCK1 and other kinases under certain experimental conditions, particularly at relatively high concentrations [79]. SB203580 also function as an activator of autophagy [80]. Although our data consistently support the involvement of MUC13/ROCK2/p38 MAPK signaling in rTsgal disrupting gut epithelial integrity, CRISPR/Cas9-mediated gene knockout, stable knockdown or rescue experiments are needed to further validate this molecular mechanism [81].

This study has certain limitations. The molecular mechanisms by which rTsgal’s direct interacts with MUC13 requires elucidation. MUC13, a mucin glycoprotein, contains possessing putative phosphorylation sites for serine and tyrosine residues. Additionally, a PKC-mediated phosphorylation has been identified within this domain [82]. Numerous structurally complicated O-glycans have been discovered in the ectodomain of MUC13; these modifying groups may include sialic acid and sulphate groups. Such glycan moieties play pivotal roles in mediating recognition events between host epithelial surfaces and invading microbial pathogens, serving as attachment sites, decoy receptors, or immunomodulatory signals [83,84]. In view of the characteristics of MUC13, it is hypothesized that rTsgal likely causes MUC13 glycosylation, thereby activating downstream signaling pathways, which requires to be confirmed in further experiments. Moreover, MUC13 has been shown to act as the receptor of rTsgal, which mediated the *in vitro* larva invasive process of gut epithelium. Although Caco-2 cells are a well-established and widely validated *in vitro* model for studying intestinal epithelial barrier, the artificial *in vitro* invasion system cannot fully recapitulate the natural intestinal environment, which includes the mucus layer, immune cell components, gut microbiota, and mechanical forces. A 2 h- incubation period primarily reflects the early invasive stage of *T. spiralis* infection rather than the complete *in vivo* invasion process. Therefore, the *in vitro* observed rTsgal disrupting tight junctions and increasing epithelial permeability in Caco-2 monolayers might reflect a potential mechanism by which *T. spiralis* facilitates larval invasion during early infection. Murine infection models with histological and molecular analyses of gut epithelial integrity will validate further the whole *in vivo* invasion dynamics and the functions of Tsgal–MUC13 interaction in *T. spiralis* infection.

In conclusions, the present study reported a previously uncharacterized mechanism by which *T. spiralis* galectin compromises intestinal epithelial barrier function and facilitates larva invasion via binding to MUC13 receptor and triggering ROCK2/MAPK signaling. These findings provide valuable insights to further elucidate the mechanisms underlying larval invasion, and to identify potential target molecules for development of novel drugs and vaccines to block *T. spiralis* infection.

## Supporting information

S1 Raw Images Raw images. (RAR)

S1 Table Specific primer sequences of human TJs and related genes for qPCR. (DOCX)

S2 Table Molecular docking scores of the interaction between rTsgal and MUC13 proteins. (DOCX)

S3 Table qPCR CT values of Figs 3–8. (XLSX)

S1 Fig The effect of rTsgal on the viability of Caco-2 cells. The rTsgal (0, 10, 20, 30, 40, 50 and 100 μg/ml) were co-incubated with Caco-2 cells for 6 and 12 h, and the cell viability was determined by CCK-8 assay. ^*^P < 0.05 compared to the DMEM group (0 μg/ml).
(TIF)

S2 Fig Confocal microscopy of rTsgal binding to Caco-2 cells. The binding sites of rTsgal with Caco-2 cells were localized in the cell membrane by confocal microscopy. Scale bars: 25 μm.
(TIF)

S3 Fig GO annotations and theoretical two-dimensional distribution of rTsgal bound-Caco-2 cell proteins. **A:** Gene ontology categories for rTsgal bound-Caco-2 cell proteins. The identified proteins were classified into cellular component, molecular function and biological process BP, CC and MF by MaxQuant according to their GO signatures. **B:** Theoretical two-dimensional (MW, pI) distribution of rTsgal bound-Caco-2 cell proteins.
(TIF)

S4 Fig IFT analyses of binding and co-localization of rTsgal and MUC13 in Caco-2 monolayer by using anti-rTsgal (green) and anti-MUC13 (red) antibodies. Yellow showed the double-stained rTsgal and MUC13. Scale bars: 25 μm.
(JPG)

S5 Fig MUC13 transcription and expression levels in Caco-2 cells after MUC13 gene was knockdown. **A:** MUC13 mRNA expression levels in Caco-2 cells transfected with two kinds of siMUC13. **B:** MUC13 protein expression levels in Caco-2 cells transfected with two kinds of siMUC13. **C:** MUC13 mRNA expression levels in Caco-2 cells transfected with three doses of siMUC13#2. **D:** MUC13 protein expression levels in Caco-2 cells transfected with three doses of siMUC13#2. **E:** MUC13 mRNA expression levels at 1–3 d after transfection with 100 nM siMUC13#2. **F:** MUC13 protein expression levels at 1–3 d after transfection with 100 nM siMUC13#2. Each assay had three replicates. ^*^P < 0.05 compared to the DMEM group.
(TIF)
