# Supplementary material for: Trichinella spiralis galectin disrupts gut epithelial integrity and mediates larval invasion via acting MUC13- ROCK2/MAPK pathway
Source: PLoS Negl Trop Dis. 2026 Sep 21;20(9):e0014684. doi: 10.1371/journal.pntd.0014684 (PMC13607845; doi:10.1371/journal.pntd.0014684)
Supplement: Supplementary file 1 [file pntd.0014684.s002.docx]

**S1 Table. Specific primer sequences of human TJs and related genes for qPCR**

| Gene names | Primer | Sequence (5′-3′) | GenBank No. |
| --- | --- | --- | --- |
| ZO-1 | Forward | CGGTCCTCTGAGCCTGTAAG | NM_001330239.4 |
|  | Reverse | GGATCTACATGCGACGACAA |  |
| E-cad | Forward | GCCTCCTGAAAAGAGAGTGGAAG | NM_131820.1 |
|  | Reverse | TGGCAGTGTCTCTCCAAATCCG |  |
| Occludin | Forward | ATGGCAAAGTGAATGACAAGCGG | XM_026274194.1 |
|  | Reverse | CTGTAACGAGGCTGCCTGAAGT |  |
| Claudin-1 | Forward | GTCTTTGACTCCTTGCTGAATCTG | NM_021101.5 |
|  | Reverse | CACCTCATCGTCTTCCAAGCAC |  |
| MUC13 | Forward | CAGACAGTGAGTCAACCACAAA | NM_033049.4 |
|  | Reverse | GGACTTGTGCTGTTTAGGGT |  |
| ROCK2 | Forward | TGGTTTCTATGGGCGAGAATGT | NM_001321643.2 |
|  | Reverse | CAAGTCGTACCTCCCTATCTGTT |  |
| GAPDH | Forward | TGTGTCCGTCGTGGATCTGA | NM_002046.7 |
|  | Reverse | TTGCTGTTGAAGTCGCAGGAG |  |
