# Supplementary material for: Trichinella spiralis galectin disrupts gut epithelial integrity and mediates larval invasion via acting MUC13- ROCK2/MAPK pathway
Source: PLoS Negl Trop Dis. 2026 Sep 21;20(9):e0014684. doi: 10.1371/journal.pntd.0014684 (PMC13607845; doi:10.1371/journal.pntd.0014684)
Supplement: Supplementary file 2 [file pntd.0014684.s003.docx]

**S2 Table. Molecular docking scores of the interaction between rTsgal and MUC13 proteins**

| Rank | rTsgal and MUC13 Docking score | M-rTsgal and MUC13  Docking score | rTsgal and M-MUC13  Docking score |
| --- | --- | --- | --- |
| Model 1 | -330.83 | -260.12 | -241.52 |
| Model 2 | -299.04 | -251.44 | -240.99 |
| Model 3 | -281.72 | -248.62 | -237.93 |
| Model 4 | -275.80 | -248.17 | -229.83 |
| Model 5 | -244.33 | -231.89 | -228.24 |
| Model 6 | -240.38 | -230.22 | -227.17 |
| Model 7 | -240.11 | -228.79 | -226.98 |
| Model 8 | -238.85 | -226.28 | -226.30 |
| Model 9 | -233.40 | -224.96 | -223.11 |
| Model 10 | -233.12 | -222.83 | -222.94 |
